# *Bifidobacterium Pseudolongum*‐Derived Acetate Attenuates Acute Pancreatitis Through GPR43‐Mediated Suppression of M1 Macrophage Polarization

**DOI:** 10.1002/advs.202517642

**Published:** 2026-03-28

**Authors:** Langyi Guan, Xueyang Li, Cong He, Pan Zheng, Yaoyu Zou, Qi Zhu, Xin Li, Jie Liang, Junmin Yang, Li Zhang, Yuman Ye, Jianhua Wan, Huajing Ke, Jianping Liu, Nonghua Lu, Nianshuang Li, Yin Zhu

**Affiliations:** ^1^ Department of Gastroenterology Jiangxi Provincial Key Laboratory of Digestive Diseases Jiangxi Clinical Research Center for Gastroenterology Digestive Disease Hospital, The First Affiliated Hospital Jiangxi Medical College Nanchang University Nanchang Jiangxi China; ^2^ Postdoctoral Innovation Practice Base The First Affiliated Hospital Jiangxi Medical College Nanchang University Nanchang Jiangxi China; ^3^ Huankui Academy Jiangxi Medical College Nanchang University Nanchang Jiangxi China

**Keywords:** acute pancreatitis, acetate, Bifidobacterium pseudolongum, GPR43, macrophage polarization

## Abstract

Acute pancreatitis (AP) is characterized by gut microbiota dysbiosis, which is marked by an expansion of pathogenic bacteria and a pronounced depletion of beneficial taxa, including *Bifidobacterium*. While *Bifidobacterium pseudolongum* (*B. pseudolongum*) possesses known probiotic properties, its potential role in protecting against AP has yet to be determined. This study shows that *B. pseudolongum* alleviates pancreatic damage, inflammation, and apoptosis in mice with caerulein‐induced and pancreatic duct ligation‐induced AP by enhancing gut barrier function and microbiota diversity. Metabolomics identifies acetate as its key metabolite, and the *ackA* gene is essential for acetate biosynthesis. Acetate supplementation protects against AP. Mechanistically, acetate acts via GPR43 to inhibit macrophage M1 polarization, and macrophage depletion abrogates this protection. Clinically, the fecal *B. pseudolongum* abundance is reduced in AP patients and is correlated with disease severity. These findings reveal a microbiota‐metabolite‐immune axis in AP and highlight the potential of *B. pseudolongum* and acetate supplementation as novel therapeutic strategies for AP.

## Graphical abstract

The severity of acute pancreatitis (AP) is linked to reduced abundance of *Bifidobacterium pseudolongum* (*B. pseudolongum*). Administration of *B. pseudolongum* or its key metabolite acetate alleviates pancreatic and systemic inflammation by suppressing M1 macrophage polarization. Mechanistically, acetate signals through the G‐protein‐coupled receptor 43 (GPR43) to restrain proinflammatory macrophage activation. These findings reveal a microbiota–metabolite–immune axis in AP and highlight the potential of *B. pseudolongum* and acetate supplementation as novel therapeutic strategies for AP.

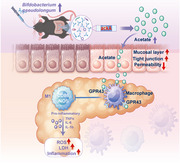

## 1 Introduction

Acute pancreatitis (AP) is an inflammatory disorder characterized by the premature activation of trypsinogen, infiltration of inflammatory cells, and acinar cell death [1, 2]. Although most cases classified as mild AP (MAP) resolve spontaneously within a week, approximately 20% of patients progress to severe AP (SAP), a condition associated with mortality rates as high as 30% [3, 4]. Currently, an effective strategy to reduce mortality in patients with SAP is unavailable.

The intestinal microbiota constitutes a key component of the mucosal barrier and plays a pivotal role in preserving intestinal homeostasis and the systemic immune balance [5]. In patients with AP, pancreatic inflammatory injury is accompanied by the excessive production of inflammatory cytokines and vasoactive substances, which exacerbate intestinal microcirculatory dysfunction and epithelial injury [6]. Increasing evidence, including our preliminary data, indicates that gut microbiota dysbiosis contributes to the progression of AP [7, 8, 9]. Disruption of the intestinal barrier facilitates bacterial translocation, thereby exacerbating both local inflammation and systemic inflammation [10]. Accordingly, modulation of the gut microbiota represents a promising therapeutic approach for AP.

During AP, the relative abundance of pathogenic bacteria, including *Escherichia coli* (*E. coli*) and *Shigella*, markedly increases, whereas the abundance of beneficial taxa such as *Bifidobacterium* significantly decreases [11]. The causal link between gut microbiota dysbiosis and AP severity has been substantiated in germ‐free animal models [9]. Consistent with these findings, a metagenomic analysis of patients with AP revealed a pronounced depletion of *Bifidobacterium* [12]. *Bifidobacterium pseudolongum* (*B. pseudolongum*), a predominant species of *Bifidobacterium*, is a gram‐positive, nonmotile, nonspore‐forming obligate anaerobe that is commonly found in the intestines of humans and other mammals [13]. Recent studies highlight its protective role as a probiotic against a range of diseases [14, 15, 16]. However, whether *B. pseudolongum* confers protective effects against AP remains to be elucidated.

Metabolites derived from specific components of the gut microbiota, including short‐chain fatty acids (SCFAs), bile acids, and tryptophan derivatives, are essential for maintaining host homeostasis by regulating epithelial barrier integrity, immune responses, and metabolic processes [17]. Among these metabolites, SCFAs are the most abundant and beneficial metabolites and are produced predominantly through the fermentation of dietary fiber and other indigestible carbohydrates by commensal bacteria [18, 19]. The principal SCFAs, acetate, propionate, and butyrate, play crucial roles in maintaining gut health. Mechanistically, SCFAs modulate host physiology by activating G protein‐coupled receptors (GPCRs), such as GPR41 and GPR43, and inhibiting histone deacetylases (HDACs) [20, 21], thereby influencing metabolic pathways, immune regulation, and cellular proliferation, particularly under inflammatory conditions [22, 23, 24].

As pivotal components of innate immunity, macrophages respond to chemotactic cues and migrate toward sites of infection, inflammation, or tissue injury, where they eliminate pathogens and debris through phagocytosis [25, 26]. Functionally, macrophages are broadly classified into two polarization states: classically activated M1 macrophages, which exhibit proinflammatory properties and secrete cytokines such as IL‐1β, IL‐6, and TNF‐α, and alternatively activated M2 macrophages, which are anti‐inflammatory and promote tissue repair through the release of cytokines, including IL‐10 and TGF‐β [27, 28]. The dynamic equilibrium between these phenotypes is a critical regulatory axis that determines whether inflammation persists or resolves under pathological conditions [29, 30]. Macrophages, neutrophils, and other immune cells interact with pancreatic acinar cells during AP, profoundly influencing the inflammatory cascade and thereby determining disease severity [31, 32]. Among these immune cells, macrophages constitute the predominant population in the pancreas during the early stage of AP. In this phase, they predominantly exhibit a proinflammatory M1 phenotype characterized by the secretion of inflammatory cytokines and mediators that drive local pancreatic inflammation. This process can further amplify inflammatory and immune responses, potentially culminating in a persistent systemic inflammatory response or multiorgan failure [33]. These observations suggest that the modulation of macrophage polarization may constitute a promising therapeutic approach for AP. However, few studies have investigated how the gut microbiota and its metabolites regulate macrophage polarization, a mechanism that may contribute to the progression of AP.

This study aimed to investigate the protective effects of *B. pseudolongum* on AP and elucidate its underlying mechanism. Our results demonstrate that *B. pseudolongum* and its metabolite acetate significantly attenuate pancreatic and intestinal injury in AP. Furthermore, we mechanistically delineated how *B. pseudolongum*‐derived acetate alleviates AP progression by modulating M1 macrophage polarization. These findings provide novel insights into the therapeutic potential of *B. pseudolongum* in AP treatment.

## 2 Results

### 2.1 *B. Pseudolongum* Protects Against AP in Two Murine AP Models

**FIGURE 1:**
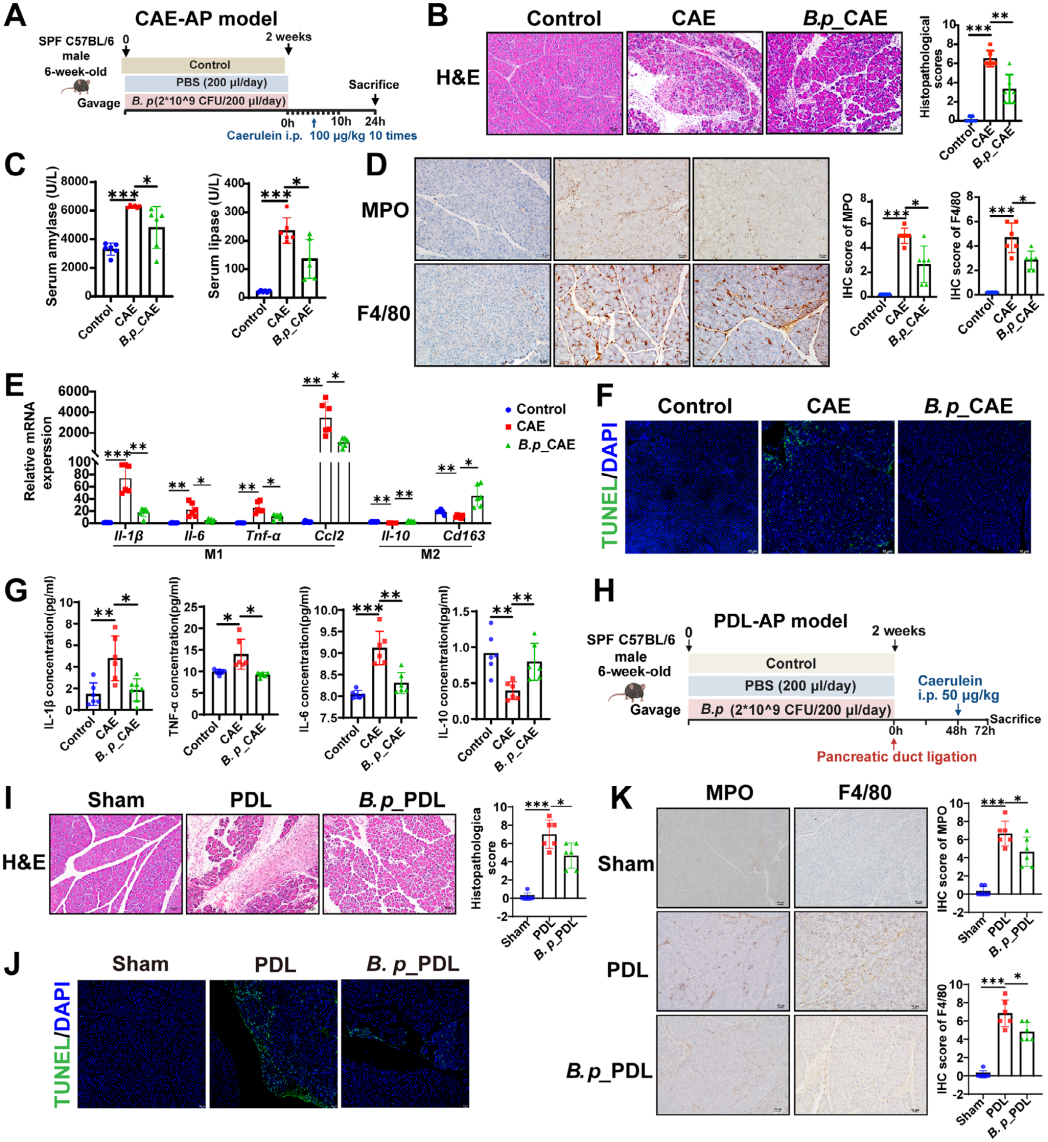
*B. pseudolongum* alleviates pancreatic injury in two mouse AP models. (A) Establishment of a CAE‐induced AP mouse model treated with *B. pseudolongum* (2×10^9^ CFUs) or PBS for two weeks. (B) Representative images of pancreatic H&E staining and histopathological scores (*n* = 6 mice/group). (C) Serum levels of amylase and lipase (*n* = 6 mice/group). (D) Representative images of IHC staining for MPO and F4/80 in pancreatic tissues and their scores (*n* = 6 mice/group). (E) Relative mRNA expression of cytokines (*Il‐1β, Il‐6, Tnf‐a, Ccl2, Il‐10* and *Cd163*) in the pancreas; *n* = 6 mice/group. (F) TUNEL staining of the pancreas (*n* = 6 mice/group). (G) Serum IL‐1β, TNF‐α, IL‐6, and IL‐10 concentrations (*n* = 6 mice/group). (H) Establishment of a PDL‐induced AP mouse model treated with *B. pseudolongum* (2 × 10^9^ CFUs) or PBS for 2 weeks. (I) Representative images of pancreatic H&E staining and histopathological scores (*n* = 6 mice/group). (J) TUNEL staining of the pancreas (*n* = 6 mice/group). (K) Representative images of IHC staining for MPO and F4/80 and their scores (*n* = 6 mice/group). Scale bar: 10 µm. The two‐sided *p* values were determined using one‐way ANOVA with Dunnett's multiple comparisons test (B‐E, G, I, K), and the data are presented as the means ± SDs. *p < 0.05, **p < 0.01, and ***p < 0.001 were considered significant. *B.p* is equivalent to *B. pseudolongum*. CAE: caerulein. IHC: immunohistochemistry. PDL: pancreatic duct ligation.

We established a mouse AP model via intraperitoneal caerulein (CAE) injection following two weeks of oral administration of *B. pseudolongum* or PBS to evaluate the protective effect of *B. pseudolongum* on AP (Figure 1A). The histopathological assessment (H&E staining) revealed attenuated pancreatic injury and inflammation in the *B. pseudolongum*‐treated CAE‐induced AP model mice compared with the PBS‐treated control mice (Figure 1B). Consistent with these findings, *B. pseudolongum*‐treated mice exhibited significantly reduced serum amylase and lipase levels (Figure 1C), as corroborated by decreased myeloperoxidase (MPO) activity and reduced F4/80‐positive macrophage infiltration (Figure 1D). Terminal deoxynucleotidyl transferase dUTP nick‐end labeling (TUNEL) staining further confirmed the decrease in pancreatic apoptosis in *B. pseudolongum*‐treated CAE‐AP mice compared with that in PBS‐treated control mice (Figure 1F). Moreover, *B. pseudolongum* administration suppressed the pancreatic mRNA expression of proinflammatory cytokines (*Il‐1β, Il‐6, Tnf‐a* and *Ccl2*), which are representative markers of M1 polarization, while increasing the expression of the M2 polarization markers *Il‐10* and *Cd163* (Figure 1E). These transcriptional changes were accompanied by decreased serum concentrations of proinflammatory cytokines and increased anti‐inflammatory cytokine levels (Figure 1G).

We subsequently established a mouse model of SAP via pancreatic duct ligation (PDL) to further substantiate the protective effect of *B. pseudolongum* on mitigating AP (Figure 1H). The histopathological evaluation revealed markedly reduced pancreatic tissue injury in *B. pseudolongum*‐treated mice, as evidenced by H&E staining, decreased MPO activity, reduced F4/80‐positive macrophage infiltration, and diminished apoptotic cell death (Figure 1I–K). These findings were consistent with those observed in the CAE‐induced model. Collectively, these results strongly support a protective effect of *B. pseudolongum* on both mild and severe forms of AP.

We employed an AP recovery model to assess the therapeutic potential of *B. pseudolongum* for established disease (Figure S1A). Histopathological analysis was used to quantify lobular integrity, acinar dedifferentiation (AD), and inflammatory cell infiltration. Compared with control mice, *B. pseudolongum*‐treated mice had significantly lower total histopathological scores, indicating attenuated disease severity (Figure S1B,C). This improvement was characterized by markedly lower scores for edema and inflammatory cell infiltration on Day 2, reflecting the mitigation of early tissue injury. Importantly, *B. pseudolongum* administration promoted later‑phase tissue repair. By Day 6 postinduction, the pancreatic architecture in the treated group was substantially restored, whereas control mice continued to display unresolved damage (Figure S1B,C).

Further analysis of mice euthanized on Day 2 revealed a distinct immunomodulatory signature associated with *B. pseudolongum* treatment. Although serum amylase and lipase levels did not differ significantly between the groups (Figure S1D), *B. pseudolongum* significantly suppressed inflammatory cell infiltration in pancreatic tissues, as evidenced by decreased MPO activity and a reduced proportion of F4/80‑positive macrophages (Figure S1E). Concurrently, serum levels of the proinflammatory cytokines IL‑1β, IL‑6, and TNF‑α were significantly lower in the *B. pseudolongum*‑treated group (Figure S1F). These findings indicate that *B. pseudolongum* exerts a multiphase therapeutic effect on AP. *B. pseudolongum* rapidly suppresses both local and systemic inflammation in the early stage and subsequently promotes the restoration of the pancreatic tissue structure in the later repair phase.

### 2.2 *B. pseudolongum* Exerts Multifaceted Protective Effects on AP by Improving Gut Barrier Integrity Through Microbiota‐Independent Mechanisms

We next examined the effects of *B. pseudolongum* on gut barrier function in AP model mice. In the experimental CAE‐induced AP mouse model, both H&E and Alcian blue‐periodic acid‐Schiff (AB‒PAS) staining revealed that *B. pseudolongum* administration markedly alleviated intestinal villus damage and goblet cell loss (Figure S2A). Consistently, Western blot analysis showed that the downregulation of the intestinal tight junction proteins Claudin‐1 and Occludin observed in AP mice was significantly reversed upon *B. pseudolongum* treatment (Figure S2B,C). Notably, mice with CAE‐induced AP exhibited increased intestinal permeability, as indicated by markedly elevated serum D‐lactate and lipopolysaccharide (LPS) levels, both of which were significantly reduced following *B. pseudolongum* administration (Figure S2D,E). Furthermore, the mRNA expression of intestinal antimicrobial peptides, including *Defa5*, *Defa21*, and *lysozyme‐1*, was significantly upregulated in *B. pseudolongum*‐treated AP mice compared with that in PBS‐treated controls (Figure S2F).

We assessed the effects of *B. pseudolongum* on the gut microbiota composition during AP by performing full‑length 16S rRNA sequencing of fecal samples from AP mice orally administered *B. pseudolongum* or PBS. Notably, *B. pseudolongum* administration significantly increased α diversity, as shown by increases in the Shannon, Chao1, and Sobs indices (Figure S2G–I). Principal coordinate analysis (PCoA) revealed a distinct microbial community structure in *B. pseudolongum*‐treated mice compared with that in PBS‐treated AP mice (Figure S2J). Both the heatmap and linear discriminant analysis effect size (LEfSe) revealed a substantially higher relative abundance of *B. pseudolongum* in the treated group than in the control group. Furthermore, the intervention led to an increase in the abundance of beneficial bacteria such as *Akkermansia muciniphila* and *Bacteroides acidifaciens* but reduced the levels of potential pathobionts, including *Mucispirillum schaedleri* and *Vampirovibrio chlorellavorus* (Figure S2K,L). The increased colonization of *B. pseudolongum* was further validated by qRT‒PCR (Figure S2M).

A germ‐free (GF) mouse model was employed, and GF mice were colonized with *B. pseudolongum* for two weeks before AP induction. The histopathological analysis showed significantly reduced pancreatic pathological scores and inflammatory cell infiltration, including decreased MPO activity and downregulated expression of the macrophage marker F4/80 (Figure S3A). These changes were accompanied by decreased serum amylase and lipase levels, along with lower serum concentrations of the proinflammatory cytokines IL‑1β and TNF‑α (Figure S3B,C). Taken together, these findings definitively establish that *B. pseudolongum* exerts a direct, intrinsic protective effect on acute pancreatitis that occurs independently of interactions with the host's commensal microbiota.

### 2.3 Active *B. pseudolongum* Produces the Functional Metabolite Acetate

**FIGURE 2:**
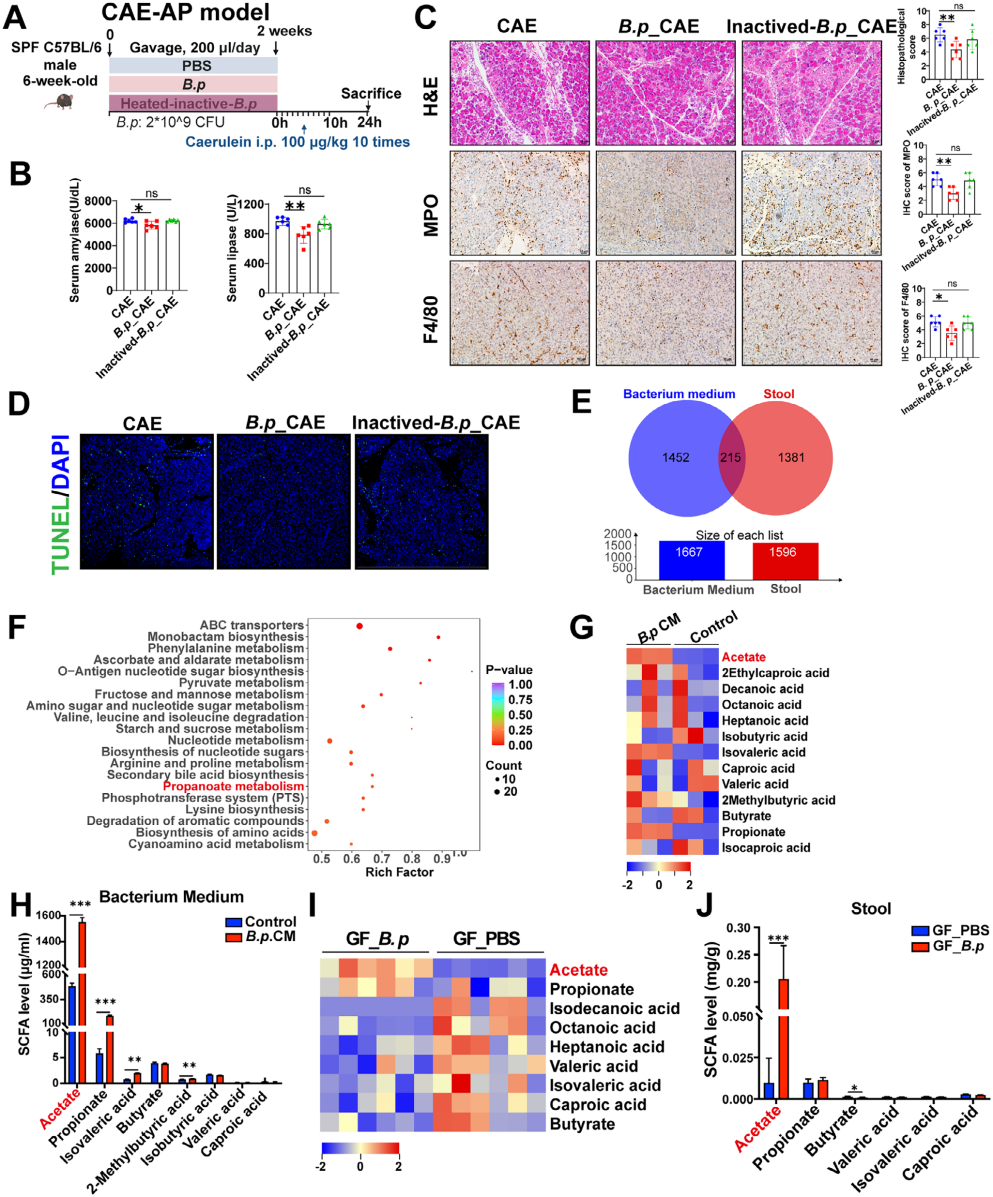
*B. pseudolongum* produces acetate as a key functional metabolite. (A) Establishment of a CAE‐induced AP mouse model with heat‐inactivated or live *B. pseudolongum*. (B) Representative images of H&E staining and IHC staining for MPO and F4/80 in pancreatic tissues and their scores (*n* = 6 mice/group). (C) Serum levels of amylase and lipase (*n* = 6 mice/group). (D) TUNEL staining of the pancreas (*n* = 6 mice/group). (E) Overlapping metabolites identified using untargeted metabolomics in *B.p* CM (*n* = 3) and germ‐free mouse feces (*n* = 6). (F) KEGG enrichment analysis of untargeted metabolomic data from *B.p* CM. (G‐H) Targeted SCFA profiling of *B.p* CM (*n* = 3 samples/group). (I, J) Targeted metabolomics analysis of SCFA levels in fecal samples (*n* = 6). Scale bar: 10 µm. The two‐sided *p* values were determined using Student's t‐test (H, J) or one‐way ANOVA with Dunnett's multiple comparisons test (B, C), and the data are presented as the means ± SDs. *p < 0.05, **p < 0.01, and ***p < 0.001. *B.p* is equivalent to *B. pseudolongum*. CAE: caerulein. IHC: immunohistochemistry. SCFAs: short‐chain fatty acids.

No *B. pseudolongum* was not detected in the pancreatic or liver tissues of either *B. pseudolongum‐gavaged* or control mice (Figure S4A,B), indicating that its ability to alleviate AP severity is unlikely to be mediated through direct bacterial translocation. Instead, the protective effect is likely attributable to soluble molecules, such as metabolites, produced by the bacterium. Accumulating evidence indicates that *B. pseudolongum* functions primarily through its small‑molecule metabolites [34, 35]. Mice were pretreated with either live or heat‐inactivated *B. pseudolongum* before the establishment of CAE‐induced AP to determine whether metabolite production contributed to its protective role in AP (Figure 2A). The administration of live *B. pseudolongum* significantly ameliorated pancreatic inflammation and injury, as evidenced by decreased serum amylase and lipase levels, improved histopathological scores, and reduced MPO activity and F4/80‐positive macrophage infiltration (Figure 2B,C). In contrast, heat‐inactivated bacteria exhibited substantially diminished protective effects in AP mice, as indicated by increased serum amylase and lipase levels, worsened histopathological scores based on H&E staining, increased MPO activity, and increased macrophage infiltration (Figure 2B,C). TUNEL staining further confirmed that heat inactivation abolished the ability of the probiotic to reduce pancreatic cell apoptosis compared with that of the live strain (Figure 2D).

Given that only viable *B. pseudolongum* conferred protective effects, we hypothesized that bioactive metabolites derived from *B. pseudolongum* may mediate its beneficial effects on AP. We performed untargeted metabolomic profiling of *B. pseudolongum*‐conditioned medium (*B.p* CM) and stool samples from germ‐free (GF) mice following *B. pseudolongum* administration to identify these metabolites. A total of 1667 metabolites in *B.p* CM and 1596 metabolites in GF mouse stool were detected, with 215 differentially expressed metabolites shared between the two sample types (Figure 2E). KEGG enrichment analysis of upregulated metabolites in *B.p* CM revealed significant enrichment in the propionate metabolism pathway (Figure 2F). Targeted SCFA profiling further demonstrated that acetate was the most markedly increased SCFA in *B.p* CM (Figure 2G,H). Consistent with these observations, fecal samples from GF mice exhibited significantly increased acetate levels (Figure 2I,J). Overall, these findings suggest that acetate, a key metabolic product generated by *B. pseudolongum*, may contribute to its protective effects of AP.

### 2.4 The ackA Gene of *B. pseudolongum* Is Critical for Acetate Production

**FIGURE 3:**
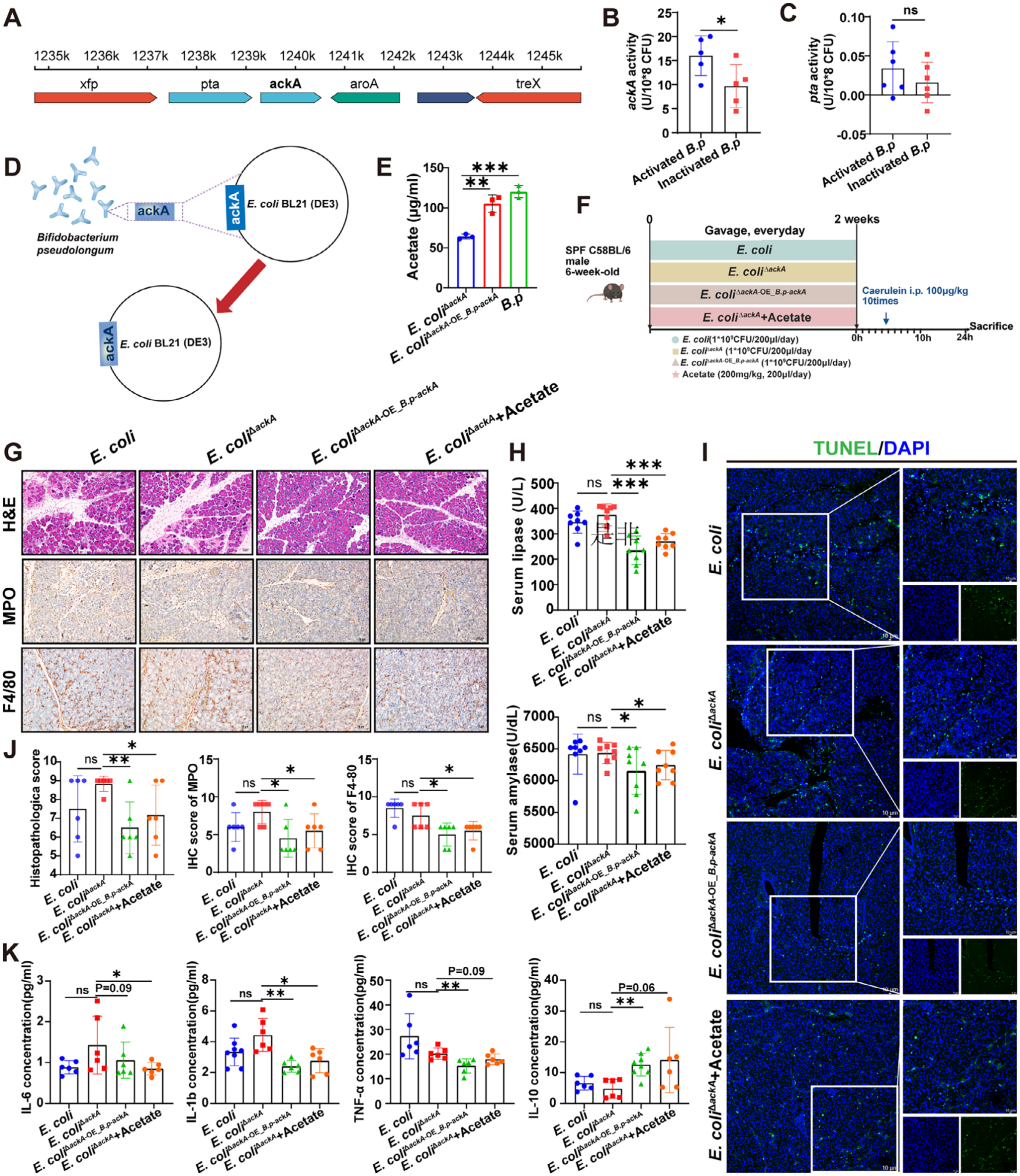
The ackA gene is critical for acetate production in *B. pseudolongum*. (A) The genome of *B. pseudolongum* contains pta and ackA genes encoding acetate‐producing enzymes. (B) The activity of ackA in live *B. pseudolongum* and inactive *B. pseudolongum* (*n* = 5 samples/group). (C) The activity of pta in live *B. pseudolongum* and inactive *B. pseudolongum* (*n* = 6 samples/group). (D) Schematic diagram of ackA gene editing in *E. coli* BL21 (DE3). (E) Acetate levels in the conditioned medium of different strains of engineered bacteria (*n* = 3 samples/group). (F) Establishment of a CAE‐induced AP mouse model with different strains of engineered bacteria. (G) Representative images of pancreatic H&E staining and IHC staining for MPO and F4/80 in pancreatic tissues and their scores (*n* = 6 mice/group). (H) Serum amylase and lipase levels (*n* = 6 mice/group). (I) Serum IL‐1β, TNF‐α, IL‐6, and IL‐10 levels (*n* = 6 mice/group). (J) TUNEL staining of the pancreas. Scale bar: 10 µm. The two‐sided *p*‐values were determined using Student's t test (B, C) or one‐way ANOVA with Dunnett's multiple comparisons test (E, H, J‒K), and the data are presented as the means ± SDs. *p < 0.05, **p < 0.01, and ***p < 0.001. *B.p* is equivalent to *B. pseudolongum*. CAE: caerulein. IHC: immunohistochemistry.

We investigated the molecular mechanism underlying acetate production in *B. pseudolongum* by performing whole‐genome sequencing. The genomic analysis identified genes encoding key enzymes involved in acetate biosynthesis, including phosphoryl acetyltransferase (*pta*) and acetate kinase (*ackA*), indicating their potential role in this process (Figure 3A). Further investigation revealed that ackA activity was significantly reduced in heat‐inactivated *B. pseudolongum* compared with viable bacteria, whereas pta activity remained unchanged (Figure 3B,C). We generated a strain of engineered *E. coli* BL21 (DE3) ΔackA (*E. coli* ^∆^ *^ackA^*) and a derivative strain overexpressing *B. pseudolongum* ackA (*E. coli* ^∆^ *^ackA^* ^‐OE_^ *^B.p^* ^−^ *^ackA^*) to directly assess the functional contribution of *ackA* to acetate production, as depicted in Figure 3D. As anticipated, the acetate levels produced by *E. coli* ^∆^ *^ackA^* ^‐OE_^ *^B.p^* ^−^ *^ackA^* were comparable to those produced by the wild‐type *B. pseudolongum* and significantly higher than those produced by the *E. coli* ^∆^ *^ackA^* control strain (Figure 3E).

We induced AP in mice via CAE injection following the administration of engineered bacterial strains and acetate supplementation to assess the role of *ackA* in acetate production and its contribution to the protective effects of *B. pseudolongum* on AP (Figure 3F). As shown in Figure 3G,H, mice treated with *E. coli* ^∆^ *^ackA^* ^‐OE_^ *^B.p^* ^−^ *^ackA^* exhibited significantly attenuated pancreatic injury and inflammation compared with those in the *E. coli* ^∆^ *^ackA^*‐treated group, as evidenced by lower histopathological scores (H&E staining), reduced MPO activity, decreased F4/80‐positive macrophage infiltration, and lower serum amylase and lipase levels. Furthermore, *E. coli* ^∆^ *^ackA^* ^‐OE_^ *^B.p^* ^−^ *^ackA^* treatment reduced pancreatic cell death and alleviated systemic inflammation, as indicated by decreased serum IL‐1β, TNF‐α, and IL‐6 concentrations (Figure 3I,J). Notably, *E. coli* ^∆^ *^ackA^* administration combined with acetate supplementation produced protective effects comparable to those observed in the *E. coli* ^∆^ *^ackA^* ^‐OE_^ *^B.p^* ^−^ *^ackA^*‐treated group, in contrast to those in the *E. coli* ^∆^ *^ackA^*‐treated group. These findings indicate that *ackA* is important for acetate production in *B. pseudolongu*m and that acetate is a key mediator of its protective effects on AP (Figure 3G–J).

### 2.5 Acetate Ameliorates Pancreatic Inflammatory Injury and Improves Gut Barrier Function in Murine AP Models

**FIGURE 4:**
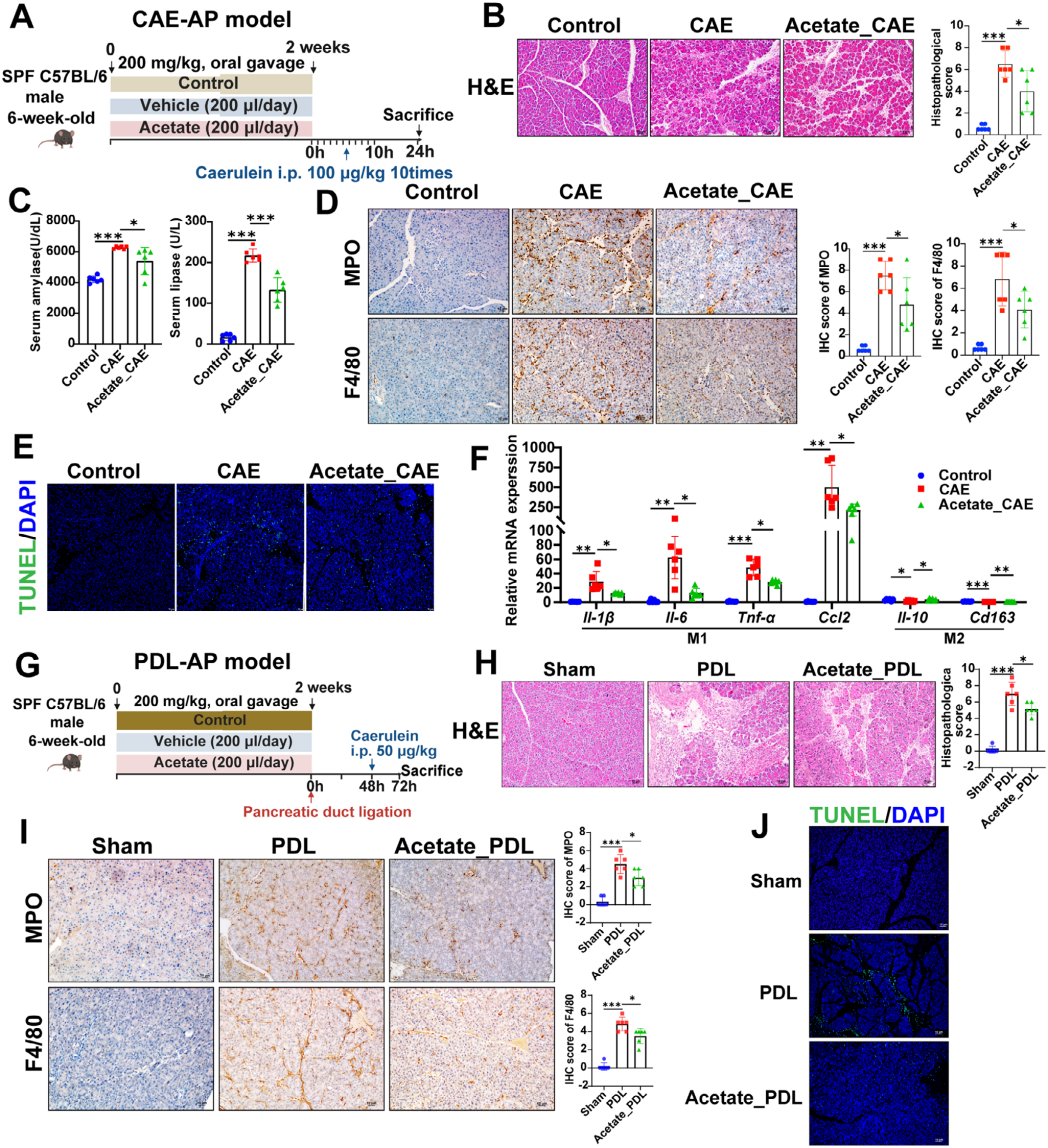
Acetate administration ameliorates AP injury in two AP mouse models. (A) Establishment of a CAE‐induced AP mouse model treated with acetate or vehicle for two weeks. (B) Representative images of pancreatic H&E staining and histopathological scores (*n* = 6 mice/group). (C) Serum amylase and lipase levels (*n* = 6 mice/group). (D) Representative images of MPO and F4/80 IHC staining (*n* = 6 mice/group). (E) TUNEL staining of the pancreas. (F) Relative mRNA expression of cytokines (*Il‐1β, Il‐6, Tnf‐a, Ccl2, Il‐10* and *Cd163*) in the pancreas (*n* = 6 mice/group). (G) Establishment of a PDL‐induced AP mouse model treated with acetate or vehicle for two weeks. (H) Representative images of pancreatic H&E staining and histopathological scores (*n* = 6 mice/group). (I) Representative images of IHC staining for MPO and F4/80 in pancreatic tissues and their scores (*n* = 6 mice/group). (J) TUNEL staining of the pancreas. Scale bar: 10 µm. The two‐sided *p* values were determined using one‐way ANOVA with Dunnett's multiple comparisons test (B–D, F, H, I), and the data are presented as the means ± SDs. *p < 0.05, **p < 0.01, and ***p < 0.001. CAE: caerulein. IHC: immunohistochemistry. PDL: pancreatic duct ligation.

Given that acetate is a key functional metabolite produced by *B. pseudolongum*, we next evaluated its therapeutic potential in AP. Mice with CAE‐ or PDL‐induced AP were orally gavaged with acetate or the vehicle control daily (Figure 4A,G). In the CAE‐AP model, acetate administration markedly reduced pancreatic injury, as evidenced by lower histopathological scores and decreased serum amylase and lipase levels (Figure 4B,C). Acetate‐treated mice also exhibited diminished pancreatic cell death, decreased infiltration of neutrophils and macrophages (Figure 4D,E), reduced expression of M1 macrophage biomarkers (*Il‐1β, Il‐6, Tnf‐a*, and *Ccl2)*, and increased expression of the M2 macrophage biomarkers *Il‐10* and *cd163* (Figure 4F). These protective effects were recapitulated in the PDL‐induced AP model, in which acetate administration significantly reduced histopathological damage, inflammatory cell infiltration, and cell death compared with vehicle treatment (Figure 4H–J).

The effects of acetate on intestinal barrier function in the CAE‐induced AP model mirrored those of *B. pseudolongum*. Acetate‐treated mice exhibited significantly increased expression of the tight junction proteins Occludin and Claudin‐1 (Figure S5A,B), accompanied by improved intestinal permeability, as reflected by reduced serum LPS and D‐lactate levels (Figure S5C,D). In addition, acetate administration significantly increased the expression of intestinal mucosal antimicrobial peptides, including *Defa5*, *Defa21*, and *Lysozyme‐1*, compared with vehicle treatment (Figure S5E). Histological analyses and AB‐PAS staining further revealed reduced intestinal epithelial damage and goblet cell loss in acetate‐treated mice (Figure S5F). Taken together, these findings indicated that acetate is a key functional metabolite of *B. pseudolongum*, protecting against both pancreatic and intestinal injury in AP mice.

### 2.6 Acetate Attenuates AP Progression via GPR43

**FIGURE 5:**
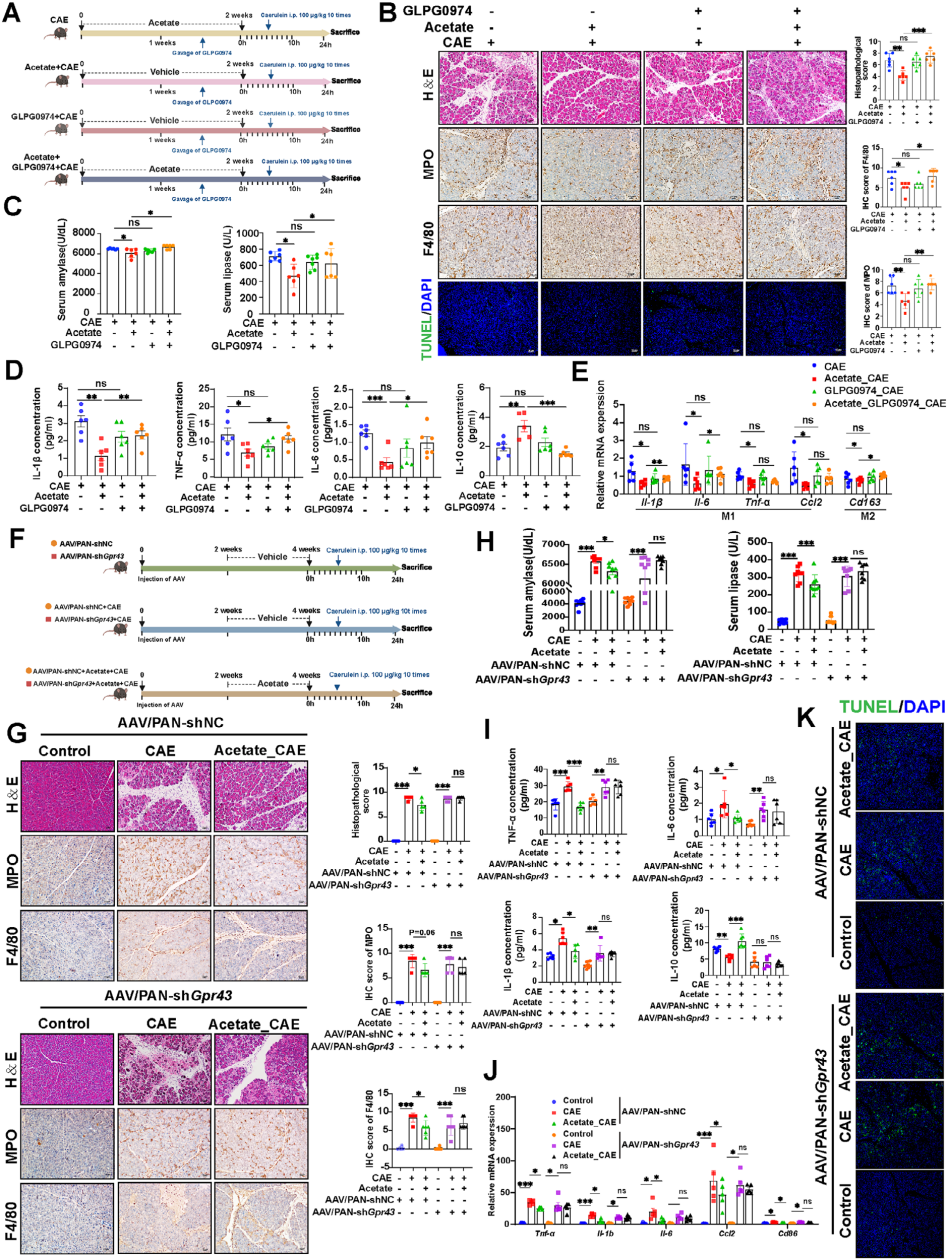
GPR43 mediates the protective effects of acetate on AP. (A) Establishment of a CAE‐induced AP mouse model treated with GLPG0974. (B) Representative images of pancreatic H&E staining, IHC staining for MPO and F4/80, and TUNEL staining in the CAE‐AP model mice treated with GLPG0974 and their scores (*n* = 6 mice/group). (C) Serum levels of amylase and lipase (*n* = 6 mice/group). (D) Serum IL‐1β, TNF‐α, IL‐6, and IL‐10 levels in the CAE‐AP model mice treated with GLPG0974 (*n* = 6 mice/group). (E) Relative mRNA expression of cytokines (*Il‐1β, Il‐6, Tnf‐a, Ccl2* and *Cd163*) in the pancreas of the CAE‐AP model mice treated with GLPG0974 (*n* = 6 mice/group). (F) Schematic diagram of the CAE‐AP model mice with pancreatic‐specific *Gpr43* knockdown (AAV/PAN‐*shGpr4*3). (G) Representative images of pancreatic H&E staining, IHC staining for MPO and F4/80 in the CAE‐AP model mice injected with AAV/PAN‐sh*Gpr43*, and their scores (*n* = 6 mice/group). (H) Serum amylase and lipase levels in the CAE‐AP model mice expressing AAV/PAN‐sh*Gpr43* (*n* = 7‐8 mice/group). (I) Serum IL‐1β, TNF‐α, IL‐6, and IL‐10 concentrations in the CAE‐AP model mice injected with AAV/PAN‐sh*Gpr43* in the pancreas (*n* = 6). (J) Relative mRNA expression of cytokines (*Il‐1β, Il‐6, Tnf‐a, Ccl2*, and *Cd163*) in the pancreas of CAE‐AP model mice injected with AAV/PAN‐sh*Gpr43* (*n* = 5 mice/group). (K) TUNEL staining of the pancreas in the CAE‐AP model mice injected with AAV/PAN‐sh*Gpr43* (*n* = 6 mice/group). Scale bar: 10 µm. The two‐sided *p* values were determined using one‐way ANOVA with Dunnett's multiple comparisons test (B–E, G–J), and the data are presented as the means ± SDs. *p < 0.05, **p < 0.01, and ***p < 0.001. CAE: caerulein. IHC: immunohistochemistry.

SCFAs exert their biological effects primarily through G protein‐coupled receptors (GPCRs) [36], among which GPR43 is a key receptor for acetate [17, 37]. We investigated the role of GPR43 in acetate‐mediated protection against AP by administering the GPR43 antagonist GLPG0974 to mice (Figure 5A). Cotreatment with GLPG0974 abolished the protective effects of acetate, resulting in aggravated pancreatic injury, elevated histopathological scores, increased infiltration of inflammatory cells, and an increased proportion of TUNEL‐positive apoptotic cells (Figure 5B). Serum amylase and lipase levels were significantly increased (Figure 5C), accompanied by increased levels of proinflammatory cytokines in both pancreatic tissue and serum and decreased levels of anti‐inflammatory cytokines (Figure 5D,E). We subsequently clarified the role of GPR43 in the signaling pathway downstream of *B. pseudolongum* using the same GPR43 antagonist, GLPG0974. Similarly, GLPG0974 abrogated the protective effects of *B. pseudolongum*, reversing the histological improvements (H&E staining) and restoring MPO and F4/80 expression (Figure S6A,B).

Given that both GPR41 and GPR43 are acetate receptors, we treated WT and *Gpr41/43*‐KO mice with acetate (Figure S7A–D). Compared with control mice, *Gpr41/43*‐KO mice exhibited significantly higher histopathological scores, higher serum amylase and lipase levels, and greater numbers of MPO‐positive neutrophils and F4/80‐positive macrophages (Figures S4G–S7E), with TUNEL staining showing consistent increases in apoptosis (Figure S7H). These results also suggest that GPR43 acts as a downstream target through which acetate ameliorates AP.

We intraperitoneally injected 8‐week‐old mice with an AAV/PAN‐sh*Gpr43* vector (three sequences; *n* = 3 mice/group; 3 × 10^11^ vg/animal) to achieve pancreas‐specific knockdown of *Gpr43* for 4 weeks to further validate the role of GPR43. Western blotting and qRT‒PCR confirmed GPR43 protein and mRNA levels (Figure S4I–K). In the CAE‐induced AP mice injected with the control AAV/PAN‐shNC, acetate administration significantly reduced pancreatic injury, neutrophil and macrophage (MPO and F4/80) numbers, and serum amylase and lipase levels; these effects were abolished by GPR43 knockdown (Figure 5F–H). Cytokine profiling showed that the acetate‐mediated suppression of IL‐1β, IL‐6, and TNF‐α expression was reversed by GPR43 knockdown, whereas serum IL‐10 levels decreased (Figure 5I,J). Moreover, GPR43 knockdown increased the number of apoptotic cells (TUNEL) in the pancreas (Figure 5K). Collectively, these results indicate that GPR43 is an essential downstream mediator of the effect of acetate on ameliorating AP.

### 2.7 Macrophages Play an Important Role in the Acetate‐Mediated Alleviation of AP

**FIGURE 6:**
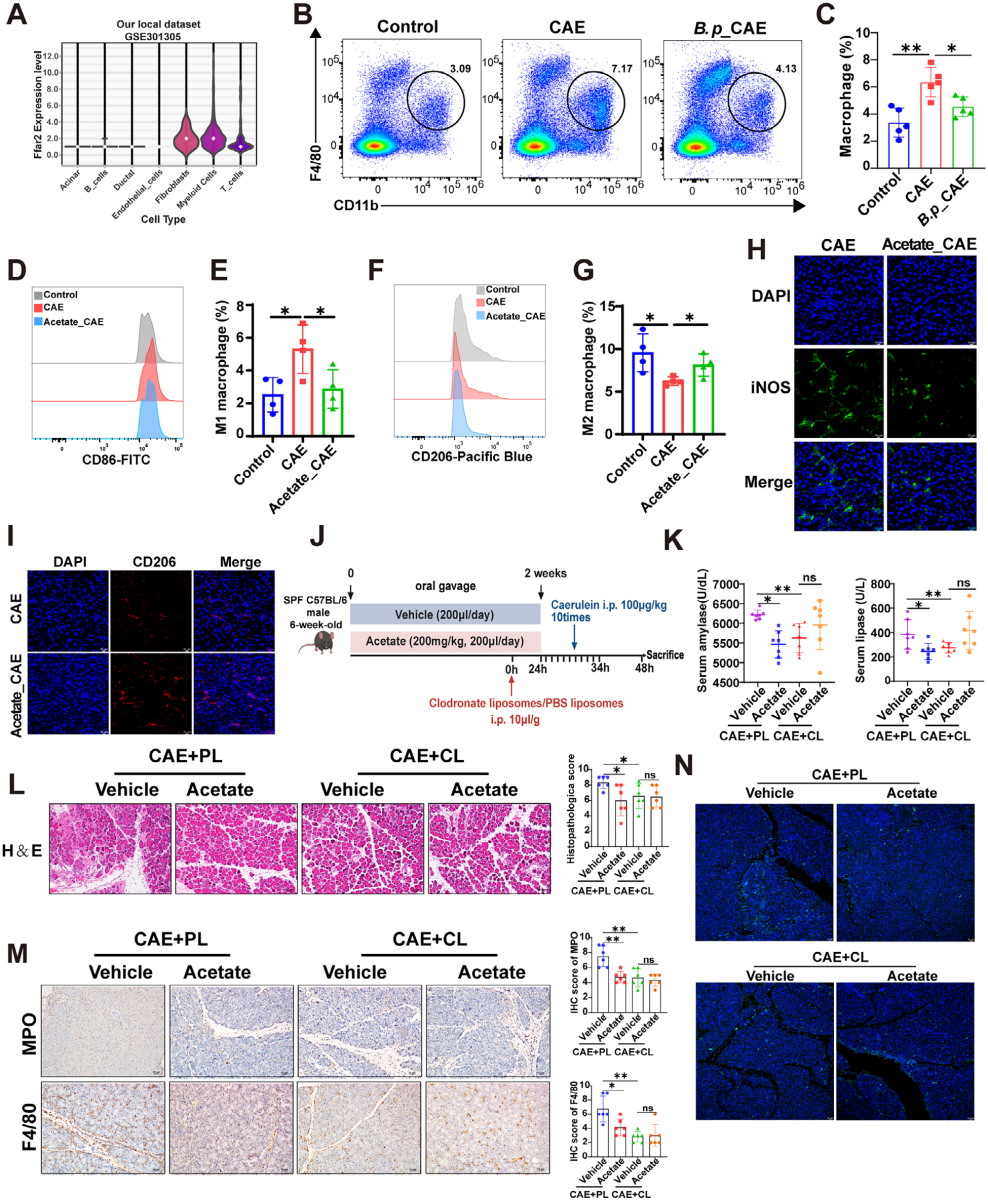
Macrophages play an important role in the acetate‐mediated alleviation of AP. (A) Violin plot of the scRNA‐seq analysis of mouse pancreatic tissues showing the expression of GPR43 (FFAR2) in different cell populations. (B, C) Flow cytometry analysis of macrophages in the spleens of *B. pseudolongum*‐treated AP mice and AP mice (*n* = 5 mice/group). (D, E) Representative histograms and percentages of M1 macrophages and M2 macrophages detected in bone marrow from the acetate‐treated group or vehicle‐treated group (*n* = 4 mice/group). (F) Immunofluorescence staining for iNOS (green) and DAPI (blue) in pancreatic tissues from mice treated with or without acetate. (G) Immunofluorescence staining for CD206 (red) and DAPI (blue) in pancreatic tissues from mice treated with or without acetate. (H) Schematic diagram of CAE‐AP model mice subjected to preadministration of clodronate‐loaded liposomes (LIPO). (I) H&E staining and histopathological score (n = 6 mice/group). (J) IHC staining for MPO and F4/80 and IHC staining scores (*n* = 6 mice/group). (K) Serum levels of amylase and lipase (n = 7 mice/group). (L) TUNEL staining of the pancreas. Scale bar, 10 µm. The two‐sided *p* values were determined using one‐way ANOVA with Dunnett's multiple comparisons test (C–E, I–K), and the data are presented as the means ± SDs. *p < 0.05, **p < 0.01, and ***p < 0.001. *B.p* is equivalent to *B. pseudolongum*. IHC: immunohistochemistry.

Macrophages are key mediators of AP progression [38]. We reanalyzed our previously published scRNA‐seq data from pancreatic tissues, which are available in the GEO database under accession number GSE301305. The single‐cell analysis of pancreatic tissues from AP and control mice revealed that GPR43 is highly expressed in myeloid cells (Figure 6A). Given the observed reduction in the number of macrophages in the pancreas of both *B. pseudolongum*‐ and acetate‐treated mice, we hypothesized that *B. pseudolongum* alleviates AP via acetate/GPR43‐mediated modulation of macrophages.

We performed flow cytometry analyses of spleens from AP mice and *B. pseudolongum*‐treated AP mice to validate this hypothesis, and the data revealed a significantly higher proportion of macrophages in AP mice than in control mice, which was markedly decreased by *B. pseudolongum* (Figure 6B,C and Figure S8A). Considering the proinflammatory role of M1‐polarized macrophages in AP [33], we next examined macrophage polarization in acetate‐treated mice. In both the bone marrow and spleen, acetate treatment reduced the proportion of macrophages and decreased M1 polarization (Figure 6D,E and Figures S8B–S8E). In bone marrow, the administration of acetate shifted the CD86‐positive macrophage peak leftward, suggesting that M1 polarization was suppressed (Figure 6D), whereas the CD206‐positive macrophage peak shifted rightward, indicating increased M2 polarization (Figure 6E). Compared with untreated AP mice, acetate‐treated AP mice exhibited decreased pancreatic iNOS expression and increased CD206 expression (Figure 6F,G), in agreement with the previous qRT‑PCR results. These findings suggest that acetate inhibits M1 polarization in macrophages.

We depleted macrophages using clodronate liposomes (LIPO) or administered control PBS‐liposomes before the establishment of the CAE‐induced AP model to directly assess the involvement of macrophages in the protective effect of acetate on AP (Figure 6H). In macrophage‐depleted mice, acetate failed to reduce pancreatic injury; serum amylase and lipase levels; or the numbers of apoptotic, neutrophil‐infiltrating, and macrophage‐infiltrating cells (Figure 6I–L). Collectively, these results confirm that acetate mitigates AP in a macrophage‐dependent manner.

### 2.8 Acetate Inhibits Macrophage M1 Polarization via GPR43 to Alleviate AP

**FIGURE 7:**
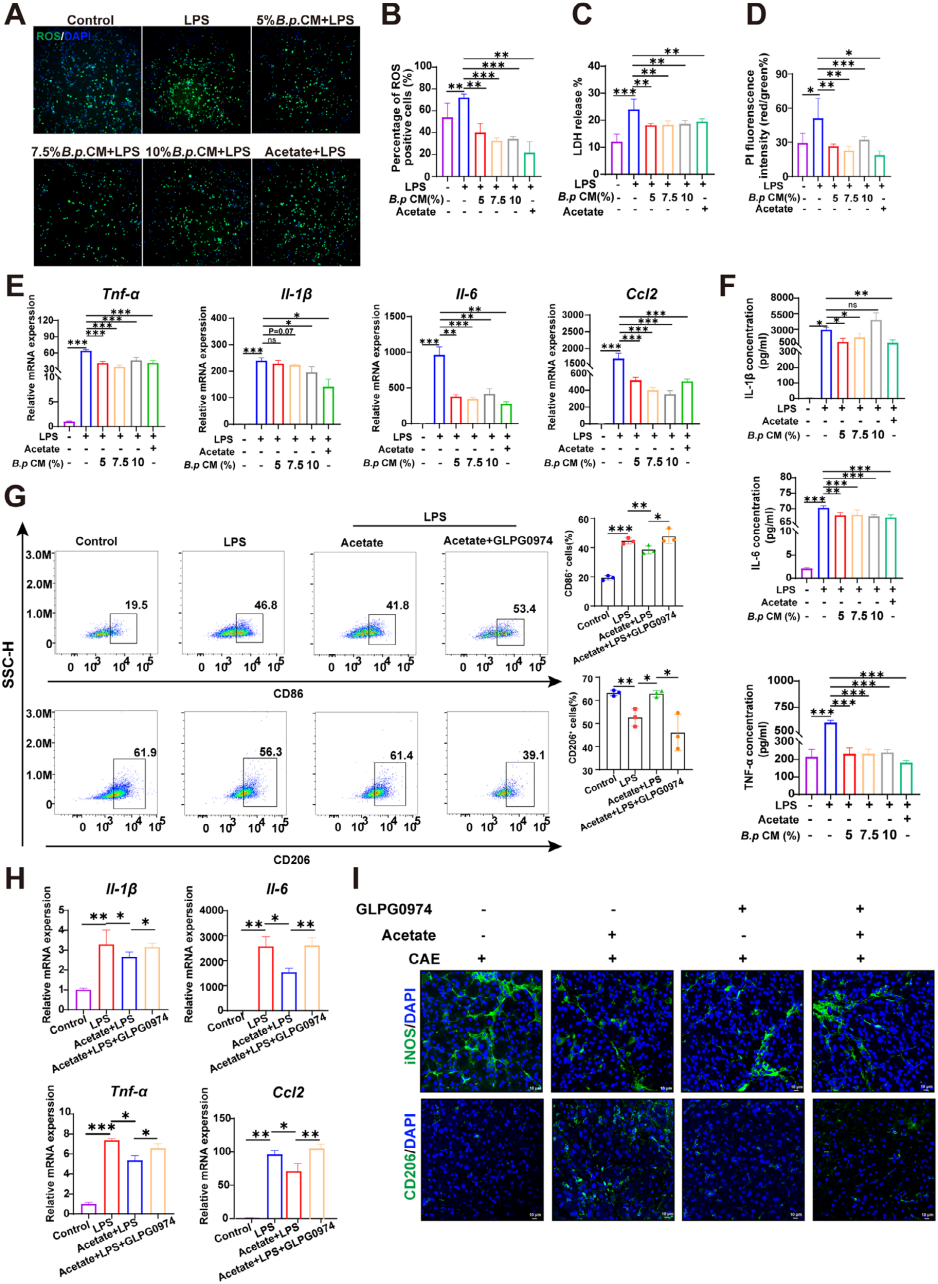
Acetate inhibits M1 macrophage polarization via GPR43 to alleviate AP. (A, B) Representative images of ROS in THP‐1 macrophages treated with LPS (1 µg/mL) and/or acetate (200 µm) or *B.p* CM (5%, 7.5%, or 10%) and the proportion of positive cells (*n* = 3 samples/group). Green, ROS; blue, DAPI. (C) Assessment of LPS‐induced macrophage damage caused by LDH release after treatment with LPS (1 µg/mL) along with acetate (200 µM) or *B.p* CM (5%, 7.5%, or 10%) (*n* = 3 samples/group). (D) Phosphatidylinositol fluorescence intensity in injured cells quantified by calcein‐AM/PI staining (*n* = 3 samples/group). (E) mRNA expression of M1 macrophage markers (*Il‐1β, Il‐6, Tnf‐a* and *Ccl2*) in THP‐1 macrophages (*n* = 3 samples/group). (F) M1 macrophage marker expression in the THP‐1 cell culture supernatant (IL‐1β, TNF‐α, and IL‐6) (*n* = 3 samples/group). (G) Flow cytometry analysis of macrophage polarization in BMDMs treated with LPS (1 µg/mL) and acetate (200 µM) or GLPG0974 (10 µm) (*n* = 3 samples/group). (H) mRNA expression of M1 macrophage markers (*Il‐1β, Il‐6, Tnf‐a*, and *Ccl2*) in THP‐1 macrophages treated with LPS (1 µg/mL) and acetate (200 µmM) or GLPG0974 (10 µ M) (*n* = 3/group). (I) Immunofluorescence staining for M1 macrophages (iNOS‐positive) and M1 macrophages (CD206‐positive) in pancreatic tissues from mice treated with GLPG0974. Scale bar: 10 µm. The two‐sided *p* values were determined using one‐way ANOVA with Dunnett's multiple comparisons test (B–F, H), and the data are presented as the means ± SDs. * < 0.05, **p < 0.01, and ***p < 0.001. *B.p is equivalent to B. pseudolongum*.

Since acetate alleviates AP via GPR43 and modulates macrophage function, we hypothesized that its protective effect occurs through the GPR43‐mediated regulation of macrophage polarization. We tested this hypothesis by cotreating PMA‐differentiated THP‐1 macrophages with LPS and either acetate or *B. pseudolongum*‐conditioned medium (*B.p* CM). As expected, acetate inhibited LPS‐induced inflammation and injury. LPS administration increased ROS production, LDH release, and cell death, all of which were markedly reduced by acetate or *B.p* CM treatment (Figure 7A–D). Moreover, *B.p* CM and acetate suppressed the LPS‐induced expression of the TNF‐α, IL‐1β, IL‐6, and CCL2 mRNAs and corresponding proteins (Figure 7E,F), indicating the suppression of M1 polarization in THP‐1 macrophages.

Bone marrow‐derived macrophages (BMDMs) from mice were isolated and subjected to flow cytometry analysis with the gating strategies shown in Figure S9A to determine whether this effect was mediated by GPR43. LPS exposure increased the proportion of CD86^+^ M1 macrophages and decreased the proportion of CD206^+^ M2 macrophages, whereas acetate treatment significantly attenuated M1 polarization and promoted M2 polarization. These effects were reversed by cotreatment with the GPR43 antagonist (Figure 7G). Similarly, in THP‐1 macrophages, the GPR43 antagonist GLPG0974 abolished the acetate‐mediated suppression of the LPS‐induced expression of proinflammatory genes (IL‐6, IL‐1b, TNF‐α, and CCL2; Figure 7H). In vivo, immunofluorescence staining of pancreatic tissue from mice with CAE‐induced AP revealed that acetate treatment significantly reduced iNOS expression (M1 marker) and increased CD206 expression (M2 marker); these effects were negated by GLPG0974 (Figure 7I). Furthermore, in BMDMs from *Gpr41/Gpr43*‐KO mice, acetate failed to suppress LPS‐induced M1 polarization, as evidenced by persistently elevated mRNA levels of *Il‐1b*, *Tnf‐α*, *Il‐6*, and *Ccl2* (Figure S9B).

We evaluated key inflammatory signaling pathways (the MAPK pathway, NF‑κB pathway, and STAT pathway) in LPS‑stimulated RAW264.7 macrophages to further elucidate the molecular mechanisms downstream of GPR43 activation. Acetate treatment significantly suppressed the LPS‑induced phosphorylation of JNK (p‑JNK), p65 (p‑p65), and STAT1 (p‑STAT1; Figure S9C). Notably, the inhibitory effect of acetate on the phosphorylation of these pathways was markedly attenuated in the presence of the GPR43‑specific antagonist GLPG0974 (Figure S9D).

Collectively, these results show that acetate acts through GPR43 to suppress macrophage M1 polarization and promote M2 polarization, an effect that is underpinned by the coordinated inhibition of the JNK, NF‑κB, and STAT1 signaling pathways, thereby alleviating AP.

### 2.9 The Abundance of *B. pseudolongum* in Fecal Samples Is Reduced During AP

**FIGURE 8:**
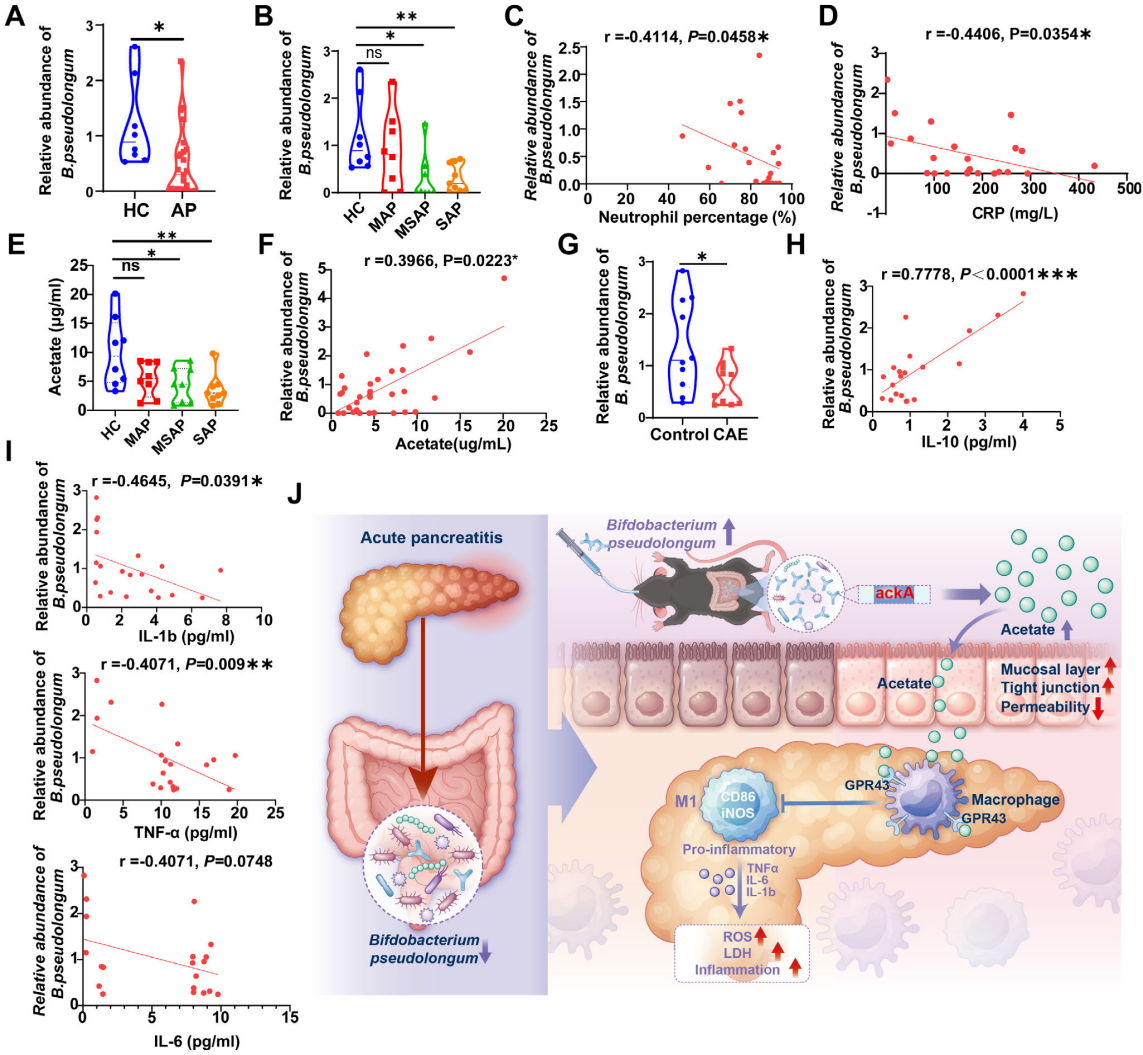
The abundance of *B. pseudolongum* in the feces is reduced in AP patients and correlates with disease severity. (A) Abundance of *B. pseudolongum* in the feces of AP patients (*n* = 24) and healthy controls (*n* = 8). (B) Abundance of *B. pseudolongum* in the feces of AP patients (MAP (*n* = 8), MSAP (*n* = 8), and SAP (*n* = 9)) compared with that in the feces of healthy controls (*n* = 8). (C, D) The abundance of *B. pseudolongum* in AP patients was negatively correlated with CRP levels and N%. (E) Fecal acetate levels in AP patients (MAP (*n* = 8), MSAP (*n* = 8), and SAP (*n* = 9)) compared with healthy controls (*n* = 8). (F) Fecal acetate levels in AP patients were positively correlated with the abundance of *B. pseudolongum*. (G) Abundance of *B. pseudolongum* in the feces of AP mice (*n* = 10) compared with control mice (*n* = 10). (H, I) The abundance of *B. pseudolongum* in AP mice was positively correlated with the IL‐10 level and negatively correlated with the levels of proinflammatory cytokines (IL‐1b, TNF‐α, and IL‐6). (J) Schematic diagram showing how *B. pseudolongum* and its metabolite acetate mitigate AP through suppression of M1 macrophages via GPR43. The two‐sided *p* values were determined using Student's t test (A, G) or one‐way ANOVA with Dunnett's multiple comparisons test (B, E), and the data are presented as the means ± SDs. R^2^ values and exact two‐sided p values calculated using Pearson's correlation analysis are shown (C, D, F, H, I). *p < 0.05, **p < 0.01, and ***p < 0.001. MAP, mild acute pancreatitis; MSAP, moderate‐to‐severe acute pancreatitis; SAP, severe acute pancreatitis; CRP, C‐reactive protein; N%, neutrophil percentage.

Fecal samples were prospectively collected from AP patients admitted to the First Hospital of Nanchang University within 72 h of symptom onset before treatment initiation and from healthy controls. The cohort included 8 healthy controls, 8 patients with MAP, 8 patients with moderate‐to‐severe AP (MSAP), and 9 patients with SAP. DNA was extracted for the quantification of *B. pseudolongum* abundance. Compared with that in healthy controls, the *B. pseudolongum* abundance was significantly reduced in AP patients, with the most pronounced decreases in MSAP (*p* < 0.05) and SAP patients (*p* < 0.01) (Figure 8A,B). The baseline characteristics are summarized in Table S1, with no significant differences among patient groups. The correlation analysis revealed that the *B. pseudolongum* abundance was negatively correlated with the neutrophil percentage (Figure 8C) and C‐reactive protein (CRP) level (Figure 8D). Furthermore, we observed significantly lower fecal acetate levels in AP patients than in controls, particularly in MSAP (*p* < 0.05) and SAP patients (*p* < 0.01) (Figure 8E). A positive correlation was also detected between the fecal acetate concentration and the relative abundance of *B. pseudolongum* (Figure 8F). In mice, the fecal *B. pseudolongum* abundance was also significantly reduced in the AP group compared with the healthy control group (Figure 8G). Moreover, the correlation analysis indicated a positive correlation between *B. pseudolongum* abundance and the levels of the anti‐inflammatory cytokine IL‐10 (Figure 8H) and a negative association with the levels of proinflammatory cytokines, including IL‐1b and TNF‐α (Figure 8I).

## 3 Discussion

Our study demonstrated that *B. pseudolongum* exerts a potential protective effect on AP primarily through the production of acetate, a process that depends on the function of the *ackA* gene. The administration of *B. pseudolongum* significantly mitigated pancreatic and intestinal injury, attenuated systemic inflammation, and preserved intestinal barrier integrity in AP mice. These effects were largely reproduced by acetate supplementation and depended on GPR43 activation, which inhibited M1 macrophage polarization.

The association between gut microbiota dysregulation and AP severity is well established, and probiotic interventions aimed at increasing the levels of beneficial microbial metabolites represent a promising therapeutic avenue [39, 40]. *Bifidobacterium*, which is among the most commonly utilized probiotics, has been shown to exert anti‐inflammatory effects on metabolic, infectious, and inflammatory conditions, including nonalcoholic fatty liver disease, influenza, and hyperlipidemia [35, 41, 42]. In AP models, *Bifidobacterium animalis* and its metabolite lactate protect against acute pancreatitis by ameliorating pancreatic and systemic inflammatory responses [43]. Here, we similarly demonstrated that *B. pseudolongum* and its metabolite acetate ameliorate AP via GPR43‐mediated regulation of macrophage polarization. Furthermore, *B. pseudolongum* administration increased tight junction protein expression and reduced intestinal permeability, underscoring its role in maintaining gut barrier function, a mechanism shared by other beneficial genera such as *Parabacteroides* and *Lactobacillus* [44].

Importantly, our findings indicate that the *B. pseudolongum* intervention reshapes the gut microbial ecosystem beyond simple compositional shifts, suggesting a restructuring of ecological interactions within the community. While the GF mouse model confirms that *B. pseudolongum* can exert protection independent of other microbes, its ecological niche and functional relationships with commensal or competitive taxa in the complex gut environment remain to be fully elucidated.

Beyond its preventive capacity, the results of the present study underscore the therapeutic promise of *B. pseudolongum* for AP. In a postonset recovery model, the administration of *B. pseudolongum* significantly increased tissue repair and inflammation resolution. Notably, this healing was accompanied by the coordinated mitigation of both local and systemic inflammatory responses, indicating that its therapeutic action is likely mediated by the same core pathway, the acetate‐GPR43‐macrophage axis, which underlies its preventive efficacy. These observations position *B. pseudolongum* as a dual‑purpose candidate for both prophylactic and therapeutic interventions in AP.

Metabolomic profiling confirmed that *B. pseudolongum* increases acetate levels both in vivo and in vitro [35]. Critically, acetate was shown to be the central mediator of the observed protective effect of *B. pseudolongum*, mitigating pancreatic inflammation, systemic cytokine release, and gut barrier disruption. We attempted to construct an *ackA*‐knockout *B. pseudolongum* strain but faced challenges with this obligate anaerobe, including strict anaerobic requirements and complex cell walls hindering plasmid delivery. Although direct genetic manipulation in *B. pseudolongum* remains challenging, heterologous expression of *B. pseudolongum ackA* in an engineered *E. coli* Δ*ackA* strain restored acetate production and resulted in the replication of the protective phenotype, confirming the essential role of *ackA* and highlighting the potential of metabolic engineering in probiotic development.

Clinically, we observed parallel reductions in fecal *B. pseudolongum* abundance and acetate levels in AP patients, with the most pronounced decreases detected in patients with moderate‑to‑severe and severe cases. The abundance of *B. pseudolongum* correlated negatively with that of systemic inflammatory markers (CRP and neutrophil percentage) and positively with the acetate concentration, mirroring our preclinical observations. These data further support the pathophysiological relevance of the *B. pseudolongum*‐acetate axis in human AP and underscore the need for longitudinal studies to delineate its temporal dynamics and causal relationship with disease outcomes.

The G protein‐coupled receptor GPR43 (FFAR2) emerged as a critical mediator of the effects of acetate in our model. This receptor, which is expressed on immune cells, enteroendocrine cells, and adipocytes, integrates metabolic and inflammatory signaling [45, 46], with known roles in regulating lipid metabolism, glucose homeostasis, and inflammation resolution in conditions such as obesity and sepsis [47, 48, 49]. Mechanistically, we further elucidated the downstream signaling events triggered by GPR43 activation. In LPS‐stimulated macrophages, acetate significantly suppressed the phosphorylation of JNK in the MAPK pathway, p65 in the NF‑κB pathway, and STAT1 in the STAT pathway. These inhibitory effects were markedly attenuated in the presence of a GPR43‑specific antagonist, establishing a multi‑pathway signaling framework through which acetate‐GPR43 engagement restrains proinflammatory macrophage polarization. This expanded mechanistic insight moves beyond a receptor‑level association to define specific downstream effectors, thereby completing the signaling cascade from the metabolite to the immune phenotype.

Macrophages undergo dynamic phenotypic switching during AP, with proinflammatory M1 macrophages dominating the early phase and M2‐like macrophages supporting subsequent tissue repair and regeneration [50, 51]. In the present study, we found that *B. pseudolongum* and acetate significantly reduced early macrophage infiltration and specifically restrained M1 polarization. These findings are consistent with previous reports that acetate can regulate macrophage accumulation and polarization to ameliorate inflammatory responses [52, 53]. Critically, we identified GPR43 as a key regulator of this process, confirming that the activation of GPR43 by acetate suppresses M1 polarization both in vitro and in vivo. Given that macrophages secrete inflammatory cytokines that contribute to acinar cell reprogramming after AP [54], our data suggest potential functional crosstalk between acetate‐modulated macrophages and pancreatic acinar cells. This interplay may constitute an additional layer of regulation in AP progression, warranting further investigation.

In this study, we clearly demonstrate that *B. pseudolongum*‐generated acetate, a functional metabolite, alleviates AP by activating GPR43 to inhibit M1 macrophage polarization through the coordinated suppression of JNK, NF‑κB, and STAT1 signaling. However, several limitations should be noted. First, while 16S rRNA gene sequencing verified an increase in the relative abundance of *B. pseudolongum* after gavage, the method is inherently unable to discriminate DNA originating from viable versus non‑viable bacteria. This limitation precludes a conclusive demonstration of persistent live colonization. Second, the inability to perform direct *ackA* knockout or overexpression in *B. pseudolongum* constitutes a technical limitation of this study. Consequently, while our heterologous expression approach supports the function of this gene, direct genetic evidence within the native bacterium would further strengthen the mechanistic conclusion regarding acetate biosynthesis. Third, while we identified several key downstream pathways, further work is needed to elucidate finer nodes within these cascades and to explore potential crosstalk.

In conclusion, this work establishes *B. pseudolongum*‑derived acetate as a key immunomodulatory metabolite that mitigates AP via the GPR43‑dependent inhibition of M1 macrophage polarization that is mechanistically linked to the inhibition of key proinflammatory signaling pathways. These findings not only elucidate a multilevel pathway linking a gut commensal bacterium to pancreatic inflammation but also highlight the translational potential of targeting the acetate‐GPR43‐macrophage axis for both the prevention and treatment of AP.

## 4 Experimental Section

### 4.1 Ethical Approval and Consent to Participate

This work was approved by the Ethics Committee of the First Affiliated Hospital of Nanchang University under protocol numbers CDYFY‐IACUC‐202306QR022 and (2024) CDYFYYLK (06‐039). All tissue samples and fecal samples were collected after obtaining informed consent.

### 4.2 Caerulein‐Induced AP

Acute pancreatitis (AP) was induced by ten hourly intraperitoneal injections of caerulein (100 µg/kg; Tocris Bioscience, UK). Control mice received equivalent volumes of saline. The mice were euthanized 24 h after the first injection.

### 4.3 Animal Models

Six‐week‐old male C57BL/6J mice (specific pathogen‐free, SPF) and germ‐free (GF) mice were purchased from GemPharmatech Co., Ltd. (Jiangsu, China). The mice were housed on a 12 h light/12 h dark cycle with ad libitum access to food and water. *Gpr41/Gpr43*‐KO mice were generated by GemPharmatech Co., Ltd. (Nanjing, China). All animal procedures were approved by the Ethics Committee of the First Affiliated Hospital of Nanchang University (CDYFY‐IACUC‐202306QR022).

In the *B. pseudolongum* preconditioning experiment, mice received daily oral gavage of *B. pseudolongum* (2×10^9^ CFUs/200 µl) or PBS for 2 weeks, followed by the induction of AP via an intraperitoneal injection of CAE or by PDL. Similarly, in the acetate preconditioning experiment, mice received daily gavage of sodium acetate (200 mg/kg in double‐distilled H_2_O) or vehicle (double‐distilled H_2_O) for 2 weeks, followed by AP induction via the injection of CAE or PDL.

For the therapeutic administration of *B. pseudolongum*, mice received intraperitoneal injections of CAE 8 times at 1 h intervals over two consecutive days to establish an AP recovery model [55], and the control group received an equivalent volume of saline. *B. pseudolongum* was administered daily from the initial modeling day until Day 6. Cohorts of *B. pseudolongum*‐treated mice and control mice were euthanized on Days 2, 4, and 6.

In the GF mouse experiment, the mice were administered *B. pseudolongum* or PBS via oral gavage every other day for two weeks. AP was subsequently induced by the intraperitoneal injection of CAE.

In the GLPG0974 intervention experiment, mice received daily oral gavage of sodium acetate (200 mg/kg in double‐distilled H_2_O) or vehicle for 2 weeks, along with daily gavage of GLPG0974 (10 mg/kg, a selective GPR43 antagonist) during the final week.

For the *Gpr41/43*‐KO experiment, the KO mice were gavaged daily with 200 mg/kg sodium acetate or vehicle for 2 weeks prior to AP induction using CAE.

We used an adeno‐associated virus carrying a CMV‐promoter‐driven shRNA targeting GPR43 (AAV/PAN‐sh*Gpr43*) to further investigate the role of GPR43 in the pancreas. For the assessment of interference efficiency in vivo, mice were injected with either AAV/PAN‐sh*Gpr43* (3 × 10^11^ vg) or a negative control vector (AAV/PAN‐shNC) and maintained for 4 weeks. During the final 2 weeks, the mice were gavaged daily with sodium acetate (200 mg/kg) or vehicle, followed by CAE‐induced AP modeling.

### 4.4 PDL‐Induced AP

The mice were anesthetized with isoflurane, and a transverse abdominal incision (∼3 cm below the left thoracic ribs) was made under a trinocular dissecting microscope (Tiannuoxiang, Beijing, CN). The liver was gently lifted using forceps to expose the junction of the duodenum and pancreas. The pancreatic duct was identified beneath a visible protrusion and ligated with nonabsorbable sutures to induce pancreatitis. The peritoneum and skin were sutured, and the wound was disinfected with iodine. Postoperative survival was monitored. CAE (50 µg/kg) was administered intraperitoneally 48 h after surgery. The mice were euthanized 72 h after the procedure.

### 4.5 Human Sample Collection

AP was diagnosed according to the *Revision of the Atlanta Classification and Definitions by International Consensus* [56]. The following exclusion criteria were applied to control for potential confounders known to influence the gut microbiota composition: (1) recent use (within three months) of antibiotics, proton pump inhibitors, or immunosuppressants; (2) a diagnosis of inflammatory bowel disease, irritable bowel syndrome, celiac disease, or other chronic gastrointestinal disorders; (3) the presence of malignancies, chronic pancreatitis, recurrent pancreatitis, or a history of major immune‑related diseases; and (4) pregnancy or lactation. A total of 25 AP patients admitted to the First Affiliated Hospital of Nanchang University within 72 h of symptom onset between October 2024 and April 2025, and 8 healthy controls were enrolled. The protocol for this study was approved by the Institutional Ethics Committee of the First Affiliated Hospital of Nanchang University (2024; CDYFYYLK (06‐039)).

### 4.6 Microbe Strains and Culture

*B. pseudolongum* (ATCC25526) was obtained from BeiNa Culture Collection Co., Ltd. (Beijing, China) and cultured in MRS medium (Hopebio, CN) at 37°C under anaerobic conditions in an AW400SG Anaerobic Workstation with a gas composition of 80% nitrogen (N_2_), 10% hydrogen (H_2_), and 10% carbon dioxide (CO_2_). The bacterial density was determined by measuring the optical density at 600 nm (OD_600_). The culture medium of *B. pseudolongum* was centrifuged at 3000 rpm for 10 min, and the supernatant was filtered through a 0.22 µm pore‐size filter to obtain *B. pseudolongum* conditioned medium (*B.p* CM). Heat‐inactivated *B.p* CM was prepared by heating *B.p* CM at 100°C for 30 min.

### 4.7 Isolation of Mouse Bone Marrow‐Derived Macrophages and Cell Culture

THP‐1 cells were cultured in RPMI‐1640 medium supplemented with 10% fetal bovine serum (FBS), 0.05 mm β‐mercaptoethanol, and 1% penicillin–streptomycin solution (Procell, Wuhan, CN). Before experimentation, the THP‐1 cells were differentiated into macrophages by treatment with 200 ng/ml phorbol 12‐myristate 13‐acetate (PMA; MedChemExpress; HY‐18739) for 48 h.

RAW264.7 cells were cultured in DMEM supplemented with 10% fetal bovine serum (FBS), 1% GlutaMAX, 1% sodium pyruvate, and 1% penicillin‒streptomycin solution (Suzhou Starfish Biotechnology Co., Ltd.).

Murine bone marrow‐derived macrophages (BMDMs) were isolated from the femurs and tibias of WT and *Gpr41/Gpr43*‐KO mice. A suspension of BMDMs was prepared by flushing the bones with PBS containing 2% FBS (Gibco, Australia) and filtering them through a sterile 70 µm cell strainer. The cells were subsequently centrifuged at 500 × g for 10 min, resuspended, and treated with red blood cell lysis buffer (Absin, Shanghai, CN) for 10 min. The isolated BMDMs were subsequently cultured in complete RPMI‐1640 medium supplemented with 10% FBS, 100 U/ml penicillin/streptomycin (NCM Biotech, Suzhou, China), and 100 ng/mL recombinant murine M‐CSF (PeproTech, USA) for 7 days. All the cells were maintained at 37°C in a humidified atmosphere with 5% CO_2_.

For LPS‐induced macrophage activation, THP‐1‐derived macrophages were stimulated with 1 µg/mL LPS and 200 µm acetate or *B.p* CM for 24 h. BMDMs were stimulated with 1 µg/mL LPS and 200 µm acetate for 6 h in the presence of 10 µm GLPG0974 to assess the role of GPR43. RAW264.7 cells were stimulated with 100 ng/mL LPS and acetate at concentrations of 0, 50, 100, or 200 µm for 24 h, with or without pretreatment with the GPR43‐specific antagonist GLPG0974 (10, 50, or 100 µm).

### 4.8 Histopathological and Immunohistochemical Analyses

Pancreatic and ileum tissues were fixed with 4% paraformaldehyde for 24 h, embedded in paraffin, and sectioned. Hematoxylin‒eosin (H&E) and Alcian blue periodic acid‐Schiff (AB‐PAS) staining (Solarbio, CN) were performed using standard protocols. Histopathological scoring was independently conducted by two professional pathologists, and the scoring was performed using a relative assessment approach within each individual model. For immunohistochemistry, the sections were deparaffinized, rehydrated, and subjected to antigen retrieval by boiling in citrate buffer for 15 min. After cooling, endogenous peroxidase activity was quenched with 3% H_2_O_2_ for 10 min. The sections were then incubated overnight at 4°C with primary antibodies against MPO (Abcam, ab208670, UK) or F4/80 (Cell Signaling Technology, Cat# 70076, USA). Following an incubation with HRP‐conjugated goat anti‐rabbit IgG, color development was achieved using a DAB staining kit (Zhongshan Biotech, Beijing, CN). MPO‐positive and F4/80‐positive cells were quantified under a light microscope (Olympus IX 70, Tokyo, Japan) and with ImageJ.

### 4.9 TUNEL and Immunofluorescence Staining

TUNEL staining was performed on paraffin‐embedded tissue sections using a Fluorescein (FITC) TUNEL Cell Apoptosis Detection Kit (Servicebio, CN) according to the manufacturer's instructions. The sections were dewaxed, rehydrated, and subjected to antigen retrieval in citrate buffer (pH 6.0). The sections were permeabilized with 0.3% Triton X‐100 for 30 min, followed by blocking with 3% BSA for 1 h. Primary antibodies against iNOS (Santa Cruz, sc‐7271) or CD206 (Abcam, ab64693) were applied and incubated overnight at 4°C. The next day, the tissue sections were washed with PBS and incubated with Alexa Fluor‐conjugated secondary antibodies (Invitrogen) for 1 h at room temperature. Nuclei were counterstained with Hoechst 33342 (ImmunoChemistry, MN, USA). Fluorescence images were acquired using a confocal microscope (Leica Stellaris 5) and analyzed using ImageJ.

The death of THP‐1 cells was assessed using propidium iodide (PI) staining with a Calcein/PI Cell Viability/Cytotoxicity Assay Kit (Beyotime, CN) according to the manufacturer's instructions. The levels of intracellular reactive oxygen species (ROS) were measured with the fluorescent probe DCFH‐DA (Thermo Fisher Scientific, C6827, USA). Briefly, the cells were washed with PBS and incubated with 10 µm DCFH‐DA at 37°C for 30 min. The nuclei were counterstained with Hoechst 33342 (Solarbio, C0031, CN). The fluorescence intensity was quantified using a high‐content analysis system (PerkinElmer, Operetta CLS&#8482, USA).

### 4.10 Western Blotting

Tissues or cells were lysed in RIPA buffer (Solarbio, R0020, CN) containing phosphatase and proteinase inhibitors at 4°C for 30 min. The protein samples were separated on 10% SDS‒PAGE gels and transferred to nitrocellulose membranes (PerkinElmer). After blocking with 5% milk, the membranes were incubated overnight at 4°C with primary antibodies against Claudin‐1 (Santa Cruz, sc‐166338), Occludin (Santa Cruz, sc‐133256), β‐actin (TransGen Biotech, #HC201‐01), p‐JNK (Cell Signaling Technology, #9251), p‐NF‑κB (Cell Signaling Technology, #9167), p‐STAT1 (Cell Signaling Technology, #3039) or GAPDH (Proteintech, 10494‐1‐AP). After being washed with PBS, the membrane was incubated with species‐matched secondary antibodies for 1 h at room temperature. The protein bands were visualized using a Bio‐Rad ChemiDoc XRS+ detection system and quantified with ImageJ, after which the target protein expression was normalized to that of GAPDH or β‐actin.

### 4.11 Biochemical Analysis

The levels of the cytokines TNF‐α, IL‐1β, IL‐6, and IL‐10 were quantified with corresponding ELISA kits from Elabscience (Elabscience Biotechnology Co., Ltd., Wuhan, CN). Serum amylase and lipase levels were measured with an α‐amylase assay (AMS) kit (C016‐1‐1) and a lipase activity assay kit (A054‐2‐1) from Nanjing Jiancheng Bioengineering Institute (Nanjing, China). A D‐Lactate Assay Kit (Abcam, ab83429, UK) and lipopolysaccharide (LPS) assay kit (Cusabio, Cat# CSB‐E09945) were used to measure the serum levels of D‐lactate and LPS.

### 4.12 Determination of Lactate Dehydrogenase (LDH) Activity

LDH levels were measured in the culture medium to determine cell damage. LDH absorbance was measured at 490 nm in accordance with the protocol approved by the manufacturer (Glpbio, Cat# GK10003, USA).

### 4.13 Quantitative Reverse‑Transcription PCR (qRT‐PCR)

Total RNA was extracted from tissue and cells using TRIzol reagent (Tiangen Biotech; China; DP424) according to the manufacturer's instructions. cDNA synthesis was performed using a FastKing cDNA First Strand Synthesis Kit (KR116; Tiangen, China). qRT‒PCR was performed on a QuantStudio 5 Real‐Time PCR System (Life Technologies) using SYBR Premix Ex Taq (RR820B; TaKaRa, Japan). The level of *B. pseudolongum* in mouse feces was also measured by qRT‒PCR. Bacterial quantitation was calculated relative to the universal 16S rRNA gene. The primers used are listed in Table S2.

### 4.14 DNA Extraction and PCR

The bacterial genomic DNA of *B. pseudolongum* was extracted with a TIANmap Bacteria DNA kit (QIAGEN, Valencia, CA). Genomic DNA was extracted from mouse feces with a DNeasy PowerSoil Kit (QIAGEN, Valencia, CA). Genomic DNA was extracted from mouse tissues with a Wizard Genomic DNA Purification Kit (Promega Corporation). PCR was performed to determine the expression of *B. pseudolongum* genes using PerfectStart Taq DNA Polymerase (TransGen Biotech). The PCR mix contained 100 ng of DNA in a final 14 µL reaction volume. PCR was performed according to the manufacturer's instructions. After the reaction, the PCR products were subjected to agarose gel electrophoresis and analysis.

### 4.15 Flow Cytometry and Antibodies

Samples of spleen tissue or blood from mice were stained with anti‐CD45 (BD Bioscience, 557659, USA), anti‐CD11b (BD Bioscience, 101212, USA), anti‐F4/80 (BD Bioscience, 101212, USA), anti‐CD86 (BD Bioscience, 159302, USA), anti‐CD206 (BD Bioscience, 141713, USA) and Fixable Viability Stain 510 (BD Bioscience, 564406, USA) antibodies to detect macrophages. After the intervention, the BMDMs were suspended in PBS for incubation. The cell suspensions were diluted to 1 × 10^6^ cells/100 µL and incubated with antibodies. The samples were stained with anti‐CD11b (BD Bioscience, 101212, USA), anti‐F4/80 (BD Bioscience, 101212, USA), anti‐CD86 (BD Bioscience, 159302, USA), and anti‐CD206 (BD Bioscience, 141713, USA) antibodies and Fixable Viability Stain 510 (BD Bioscience, 564406, USA). When samples and cells were stained, cells were acquired and detected with a NovoCyte D3000 flow cytometer (Agilent). The data were analyzed using FlowJo v.10 software (TreeStar).

### 4.16 Metabolomics Analysis

Stool (50 mg), serum (50 µL), and conditioned culture medium (50 µL) samples were processed for metabolomic profiling. Nontargeted LC‒MS/MS analysis and targeted short‐chain fatty acid (SCFA) metabolomic analysis were performed by MetWare (Wuhan, CN). Sample pretreatment was performed according to a previously described method [57].

Nontargeted LC‐MS/MS analyses were performed using a UHPLC system (Prominence Plus LC‐30A, Shimadzu, Japan) with a Waters ACQUITY Premier HSS T3 column and a TripleTOF 6600 mass spectrometer (AB SCIEX, Foster City, CA, USA). The raw data were transformed into mzXML format using ProteoWizard and then subjected to peak extraction, alignment, and retention time correction with the XCMS program. Peaks with >50% missing data in each sample group were filtered out, and blank values were imputed with the k‐nearest neighbor (KNN) algorithm. Peak area correction was performed via support vector regression (SVR), and the corrected peaks were subjected to metabolite identification by searching a laboratory‐established database, integrated public repositories, prediction libraries, and the metDNA method. The metabolomic data were analyzed using the MetaboAnalystR R package.

For the targeted SCFA metabolomic analysis using GC‒MS/MS, an Agilent 7890B‐7000D GC‒MS/MS platform was used. The qualitative analysis of the mass spectrometry data was performed using an MS database constructed with standard compounds. The quantitative analysis was conducted via the multiple reaction monitoring (MRM) mode of the triple quadrupole mass spectrometer, followed by an evaluation of the data.

### 4.17 Full‑Length 16S Ribosomal RNA (rRNA) Sequencing

Fecal samples were collected from mice in the *B. pseudolongum*‐gavaged and PBS‐gavaged groups following the establishment of the caerulein‐induced AP model. Full‑length 16S rRNA was subsequently used to detect intestinal flora in feces by Shanghai Majorbio Biopharm Technology Co., Ltd. (Shanghai, China). First, total microbial genomic DNA was extracted from the fecal samples and amplified. The DNA extract was checked on a 1% agarose gel, and the DNA concentration and purity were determined with a NanoDrop2000 spectrophotometer (Thermo Fisher Scientific, USA). The 27F (5’‐AGRGTTYGATYMTGGCTCAG‐3’) and 1492R (5’‐RGYTACCTTGTTACGACTT‐3’) primers were chosen to amplify the bacterial 16S rRNA genes. Finally, the purified products were pooled in equimolar amounts, and a DNA library was constructed using the SMRTbell prep kit 3.0 (Pacific Biosciences, CA, USA) according to the instructions of PacBio. Bioinformatics analysis of the gut microbiota was performed using the Majorbio Cloud platform (https://cloud.majorbio.com).

### 4.18 Acetate Quantification

The acetate levels in the conditioned culture supernatants of different bacteria were quantified with an acetate assay kit (Mlbio, Shanghai, CN) according to the manufacturer's instructions.

### 4.19 Detection of ACKA and PTA Activities

Acetate kinase (ACKA) and phosphotransacetylase (PTA) activities were detected using a PTA activity assay kit (Solarbio, CN) and an acetylkinase (ACK) activity assay kit (Solarbio, CN), respectively, according to the manufacturer's instructions.

### 4.20 AAV/PAN‐shGpr43

The lentivirus‐mediated shRNA expression vectors targeting murine GPR43 (target sequence #1: 5′‐GCTGTTGTGACGCTTCTTAAT‐3′; target sequence #2: 5′‐GCTGTTGTGACGCTTCTTAAT‐3′; target sequence #3: 5′‐GCTGTTGTGACGCTTCTTAAT‐3′) were purchased from OBiO Technology (Shanghai, CN).

### 4.21 Microbial Gene‐Editing Strategies

The pET‐28a (+)‐6xHis/Chain A plasmid (the DNA fragments encoding full‐length ackA were cloned and inserted into the pET28a vector) was constructed by Testobio (Ningbo, CN). *E. coli* BL21 (DE3) cells transfected with plasmids were grown on LB agar (Thermo Fisher Scientific, USA) and LB agar supplemented with 50 µg/mL kanamycin (K8020; Solarbio, CN) overnight at 37°C to generate a *B. pseudolongum* ackA‐overexpressing (OE) strain. For bacterial gavage, the bacterial culture medium was centrifuged at 4000 g at 4°C for 10 min, after which the pellets were suspended in PBS. For bacterial colonization, the mice were divided into four groups: (1) *E. co*li BL21 (DE3), (2) *E. coli* BL21 (DE3) ΔackA, (3) *E. coli* BL21 (DE3) Δ*ackA‐overexpressing B. pseudolongum ackA* (*E. coli* ^∆ackA‐OE_^ *^B.p^* ^‐ackA^), and (4) *E. coli* BL21 (DE3) Δ*ackA* along with acetate treatment. Mice were treated with 2 × 10^8^ CFUs of different strains with or without acetate in 200 µl of sterile PBS by gavage for 2 consecutive weeks followed by the induction of AP.

### 4.22 Statistical Analyses

All the statistical analyses were performed using SPSS 26.0 software and GraphPad Prism 9.0. The data are presented as the means ± standard deviations (means ± SDs) from three independent experiments. Differences between the two groups were compared using Student's t‐test. For comparisons among three or more groups, one‐way ANOVA followed by Dunn's correction was employed. Spearman's rank correlation coefficient was calculated for the correlation analyses. A *p* value of less than 0.05 was considered to indicate statistical significance.

## Author Contributions

Y.Z. and N.S.L. designed the experiments. L.Y.G., X.Y.L., and C.H. conducted the experiments. P.Z., Y.Y.Z., Q.Z., X.L., J.L., J.M.Y., L.Z., Y.M.Y., H.J.K., and J.H.W. examined and analyzed the data. L.Y.G., X.Y.L., and N.S.L. drafted the paper. J.P.L., NH.L., N.S.L., and Y.Z. revised the manuscript. All the authors read and approved the final manuscript.

## Conflicts of Interest

The authors declare no conflicts of interest.

## Supporting information




**Supporting file 1**: advs75008‐sup‐0001‐SuppMat.docx

**Supporting file 2**: advs75008‐sup‐0002‐Table S1.xlsx

**Supporting file 3**: advs75008‐sup‐0003‐Table S2.docx

**Supporting file 4**: advs75008‐sup‐0004‐Table S3.xlsx

## Data Availability

The metabolomic data reported in this paper have been deposited in the OMIX, China National Center for Bioinformation/Beijing Institute of Genomics, Chinese Academy of Sciences (https://ngdc.cncb.ac.cn/omix, under accession no. OMIX011373). The 16S rDNA sequencing data reported in this paper have been deposited in the SRA database (https://www.ngbi.nlm.nih.gov/sra/PRJNA1302438, BioProject ID: PRJNA1302438).
