# Supplementary material for: Bifidobacterium Pseudolongum‐Derived Acetate Attenuates Acute Pancreatitis Through GPR43‐Mediated Suppression of M1 Macrophage Polarization
Source: Adv Sci (Weinh). 2026 Mar 28;13(32):e17642. doi: 10.1002/advs.202517642 (PMC13252603; doi:10.1002/advs.202517642)
Supplement: Supplementary file 1 — Supporting file 1: advs75008‐sup‐0001‐SuppMat.docx [file ADVS-13-e17642-s001.docx]

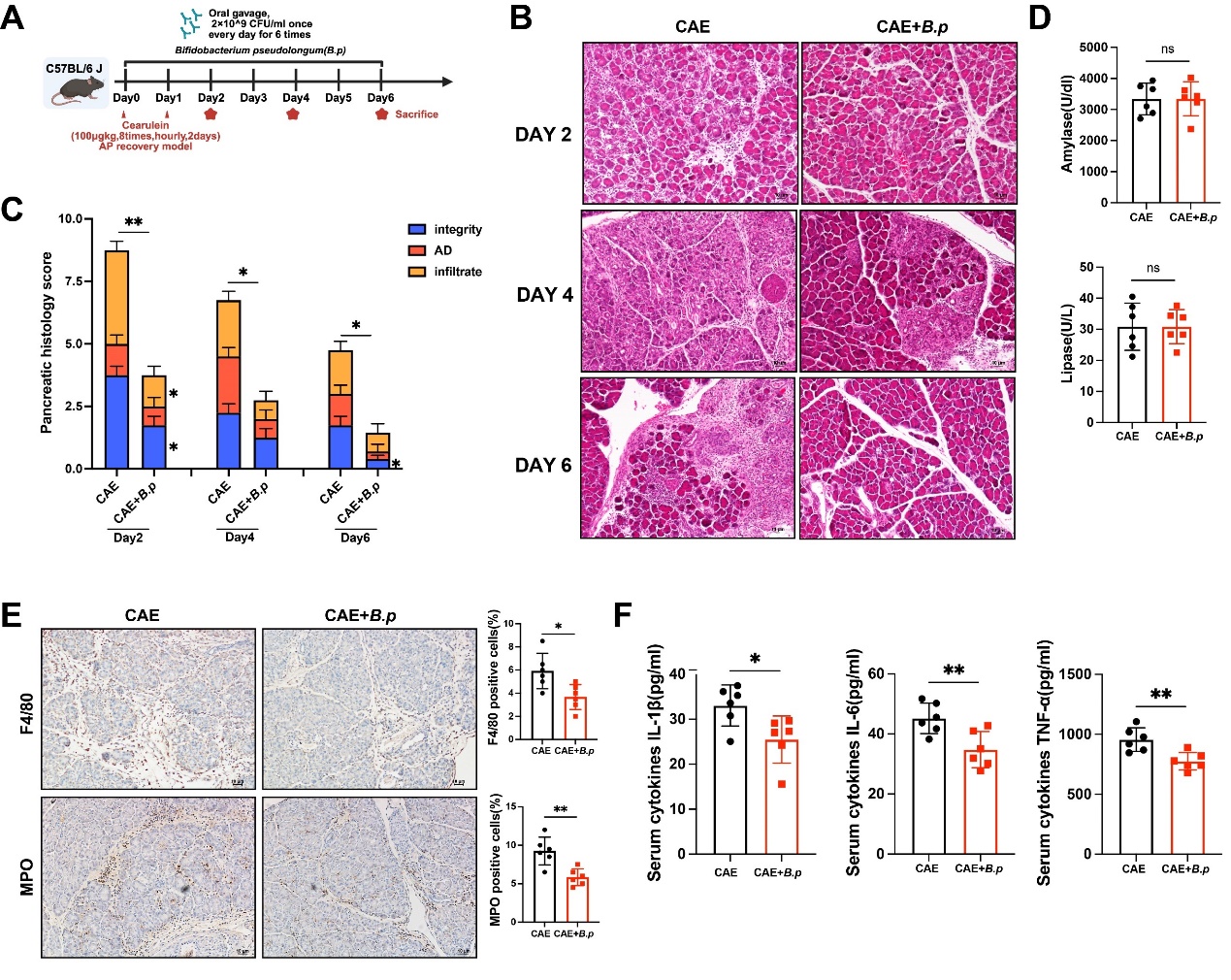


**Figure S1: The therapeutic administration of *B. pseudolongum* alleviated CAE-induced AP.** (A) A schematic diagram illustrating the construction of the AP recovery model and the timeline for *B. pseudolongum* administration. The mice were sacrificed on Days 2, 4, and 6 following AP induction. (B–C) Representative images of H&E-stained pancreatic tissues on Days 2 (n=6), 4 (n=6) and 6 (n=6) and their histopathological scores. (D) Serum amylase and lipase levels (n=6 mice/group). (E) Representative images of IHC staining for MPO and F4/80 and their IHC staining scores (n=6 mice/group). (F) Serum IL-1β, IL-6 and TNF-α levels (n=6 mice/group). Scale bar: 10 μm. The two-sided p values were determined using Student’s t test (C–F), and the data are presented as the means ± SDs. **P* < .05, ***P* < .01, and ****P* < .001 were considered significant. *B.p* is equivalent to *B. pseudolongum.* CAE: caerulein. AD: acinar dedifferentiation. IHC: immunohistochemistry.
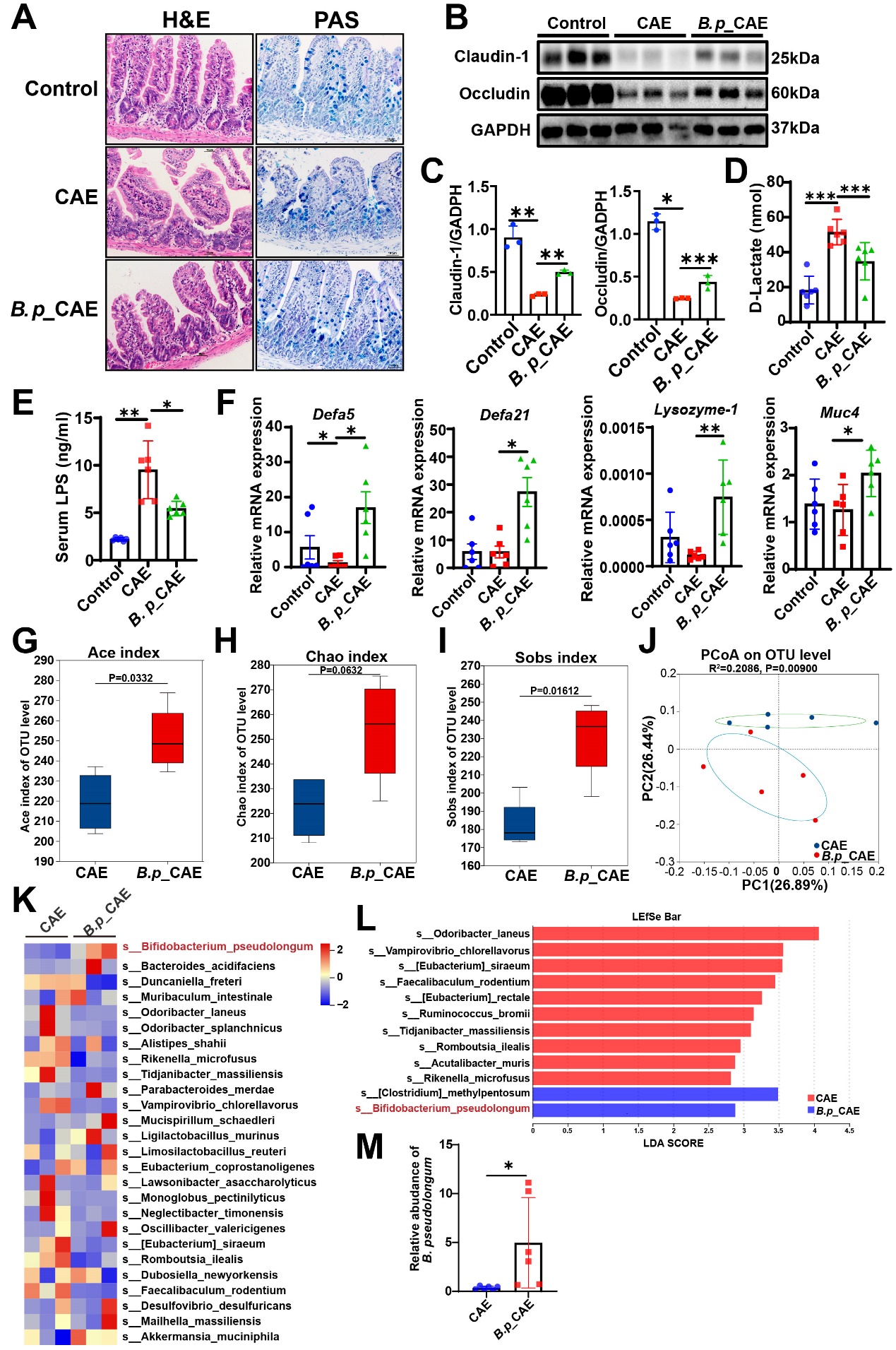


**Figure S2: *B. pseudolongum* modulates gut barrier integrity and the gut microbiota composition in the CAE-induced AP model.** (A) Representative images of intestinal H&E staining and AB-PAS staining. (B–C) Western blot analysis of intestinal tight junction proteins (Occludin and Claudin-1) and quantification of protein expression (n=3). (D) Serum D-lactate levels (n=6). (E) Serum LPS levels (n=6). (F) Relative mRNA expression of *Defa5*, *Defa21*, *lysozyme-1* and *Muc4* in the intestine (n=6). (G) Ace index (H), Chao index and (I) Sobs index calculated using full‑length 16S rRNA sequencing data from *B. pseudolongum*-treated AP mice and PBS-treated AP mice. (J) Principal coordinate analysis (PCoA) (n=5). (K–L) Heatmap and LEfSe of the differential abundance of the gut microbiome based on full‑length 16S rRNA sequencing (n=3), (M) relative abundance of *B. pseudolongum* in the fecal samples from *B. pseudolongum*-treated AP mice and PBS-treated AP mice detected using qRT‑PCR (n=6). Scale bar: 10 μm. The two-sided p values were determined using Student’s t test (M) or one-way ANOVA with Dunnett’s multiple comparisons test (C–F), and the data are presented as the means ± SDs. **P* < .05, ***P* < .01, and ****P* < .001 were considered significant. *B.p* is equivalent to *B. pseudolongum.* CAE: caerulein.


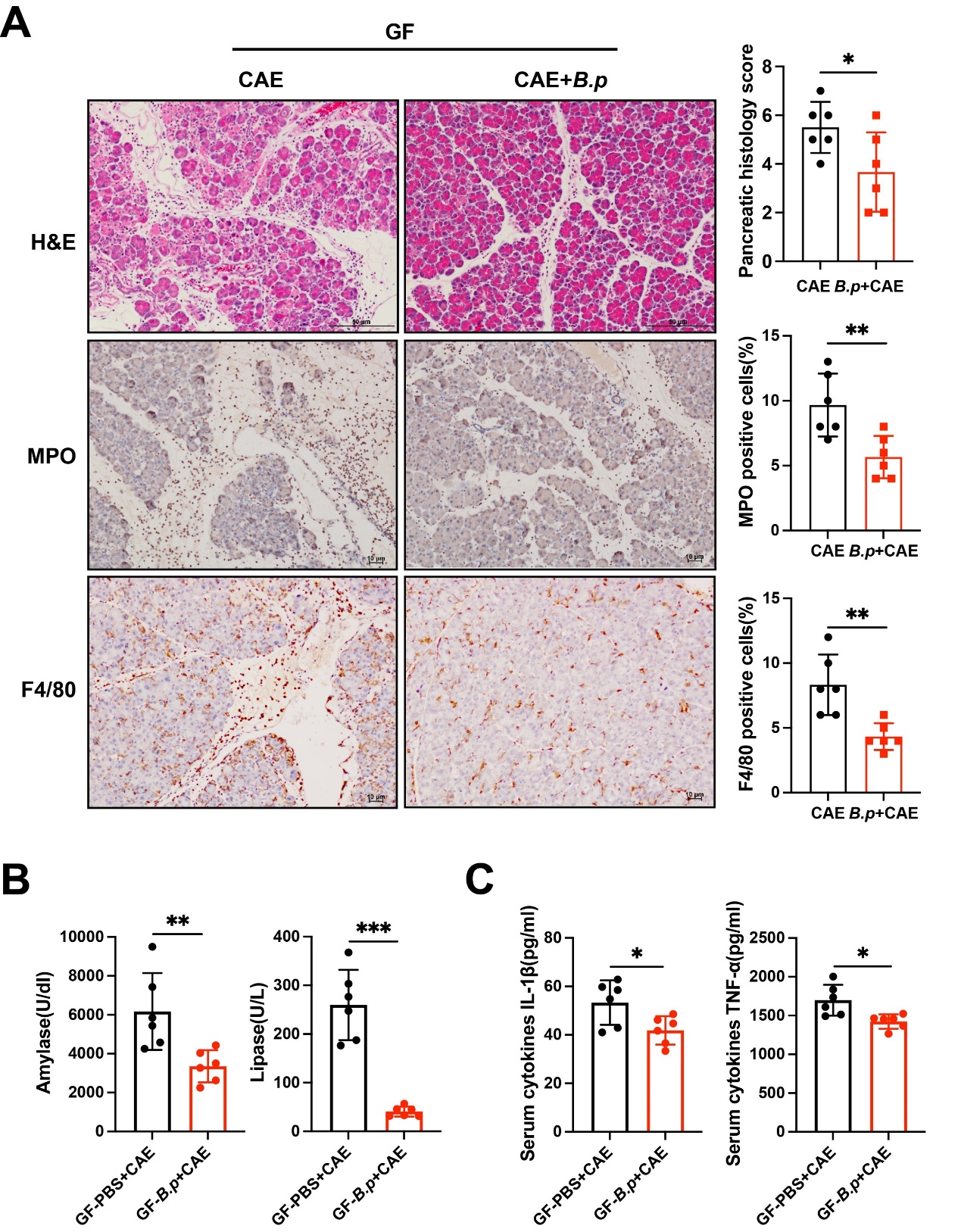


**Figure S3:** ***B. pseudolongum* attenuates pancreatic injury and inflammation in the CAE-induced AP model established in a germ-free mouse model.** (A) Representative images and quantitative scores of H&E staining and immunohistochemistry for MPO and F4/80 in pancreatic tissues (n=6 mice per group). (B) Serum levels of amylase and lipase. (C) Serum concentrations of the proinflammatory cytokines IL‑1β and TNF‑α. Scale bar: 10 μm. CAE, caerulein; GF, germ‑free; IHC, immunohistochemistry. The two-sided p values were determined using Student’s t test (A–C), and the data are presented as the means ± SDs. **P* < .05, ***P* < .01, and ****P* < .001 were considered significant. **GF: germ‑free.**

**
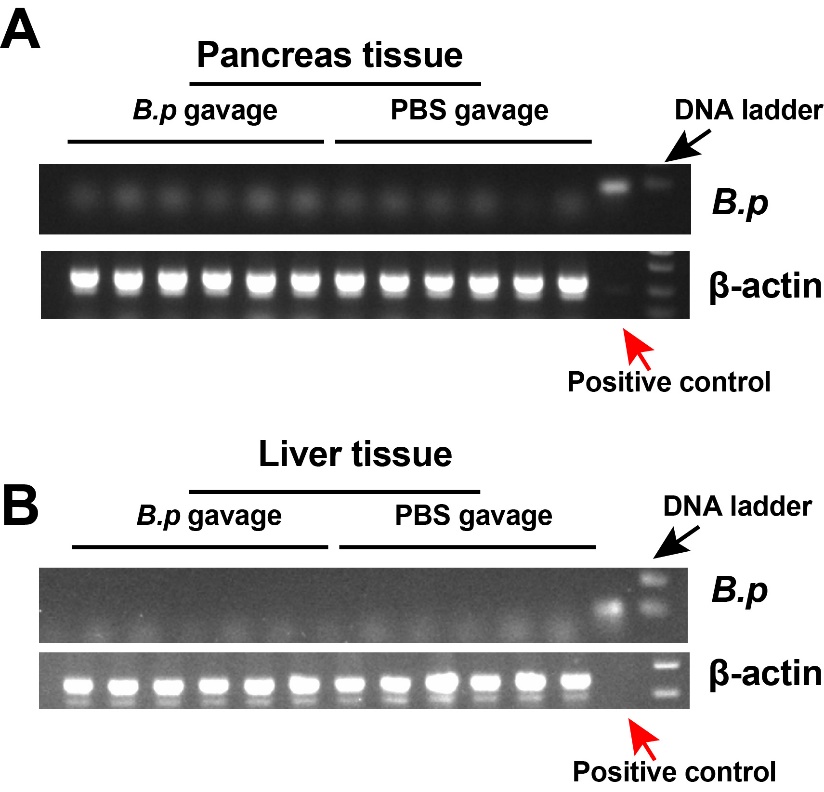
**

**Figure S4: Detection of *B. pseudolongum* in tissues by PCR.** (A) *B. pseudolongum* was not detected in the pancreas of *B. pseudolongum*-gavaged (n=6) or PBS-gavaged (n=6) mice by PCR. (B) *B. pseudolongum* was not detected in the livers of *B. pseudolongum*-gavaged mice by PCR.


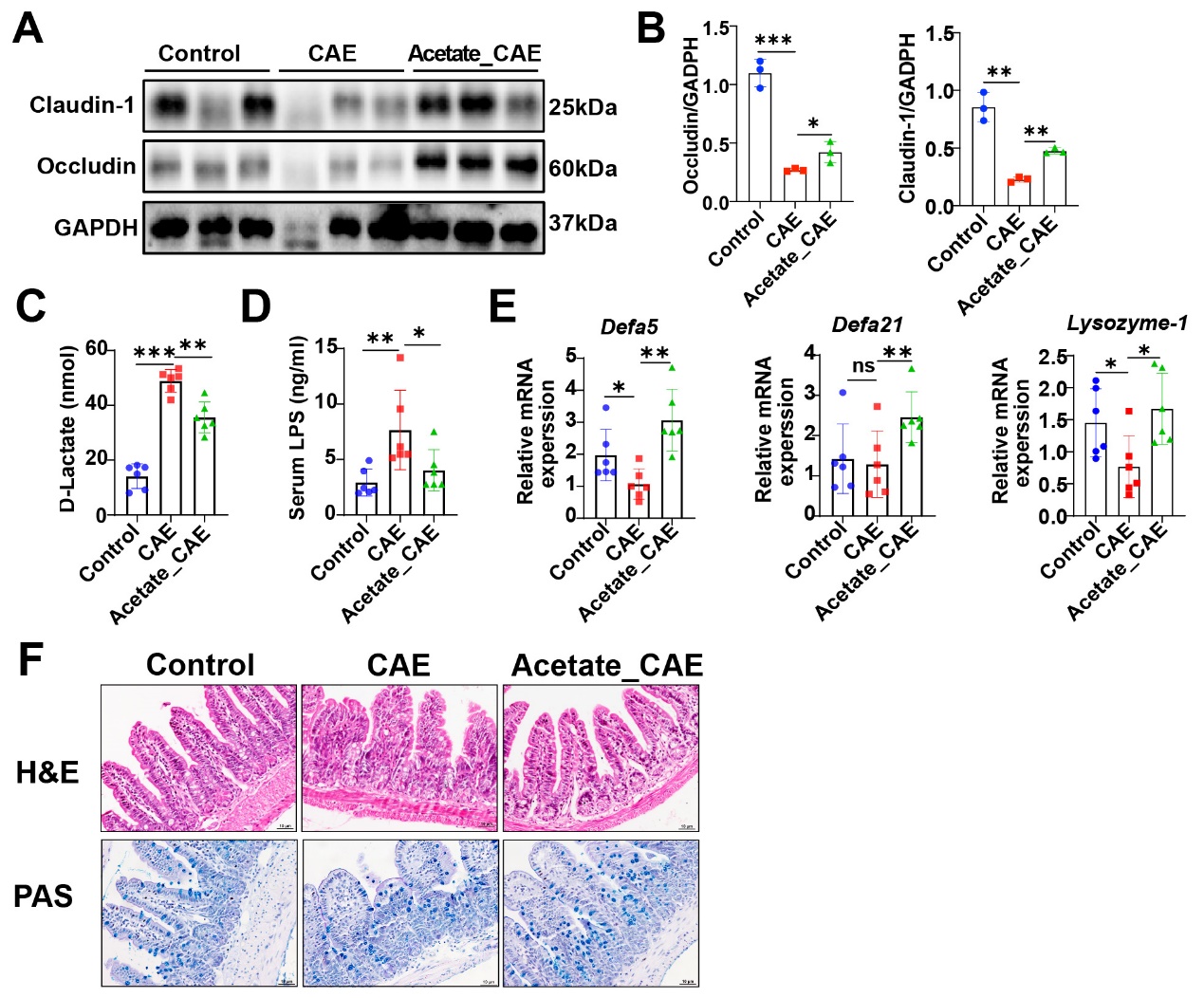


**Figure S5: Acetate improves gut barrier function in mice with CAE-induced AP.** (A–B) Western blot analysis of intestinal Occludin and Claudin-1 expression and the quantitative results (n=3). (C) Serum D-lactate levels and (D) serum LPS levels (n=6). (E) Relative mRNA expression of *Defa5*, *Defa21* and *lysozyme-1* in the intestine (n=6)*.* (F) Representative images of intestinal H&E staining and AB-PAS staining. Scale bar: 10 μm. The two-sided p values were determined using one-way ANOVA with Dunnett’s multiple comparisons test (B–E), and the data are presented as the means ± SDs. **P* < .05, ***P* < .01, and ****P* < .001. CAE: caerulein.


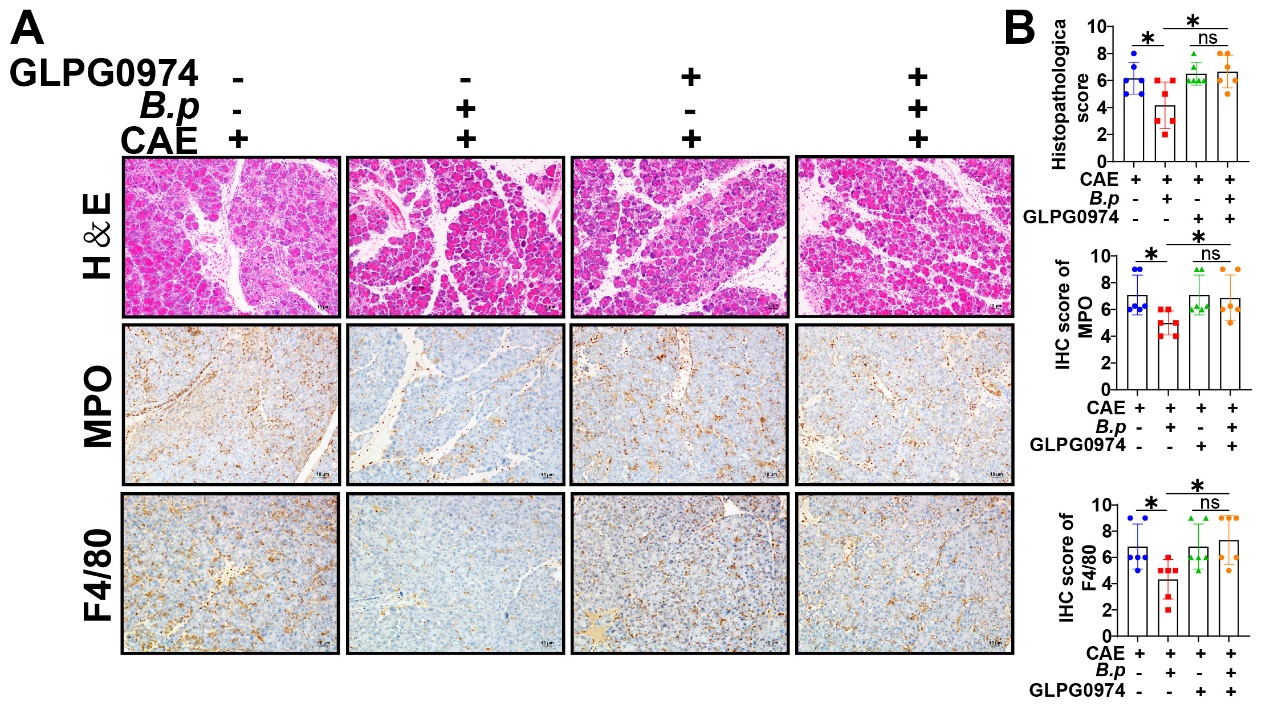


**Figure S6: GPR43 significantly affects the *B. pseudolongum*-mediated alleviation of AP.** The mice were treated with *B. pseudolongum* and GLPG0974 following an intraperitoneal injection of CAE. (A) Representative images of pancreatic H&E staining and IHC staining for MPO and F4/80. (B) Histopathological scores and IHC staining scores. Scale bar: 10 μm. The two-sided p values were determined using one-way ANOVA with Dunnett’s multiple comparisons test (B), and the data are presented as the means ± SDs. **P* < .05, ***P* < .01, and ****P* < .001. CAE: caerulein. IHC: immunohistochemistry.


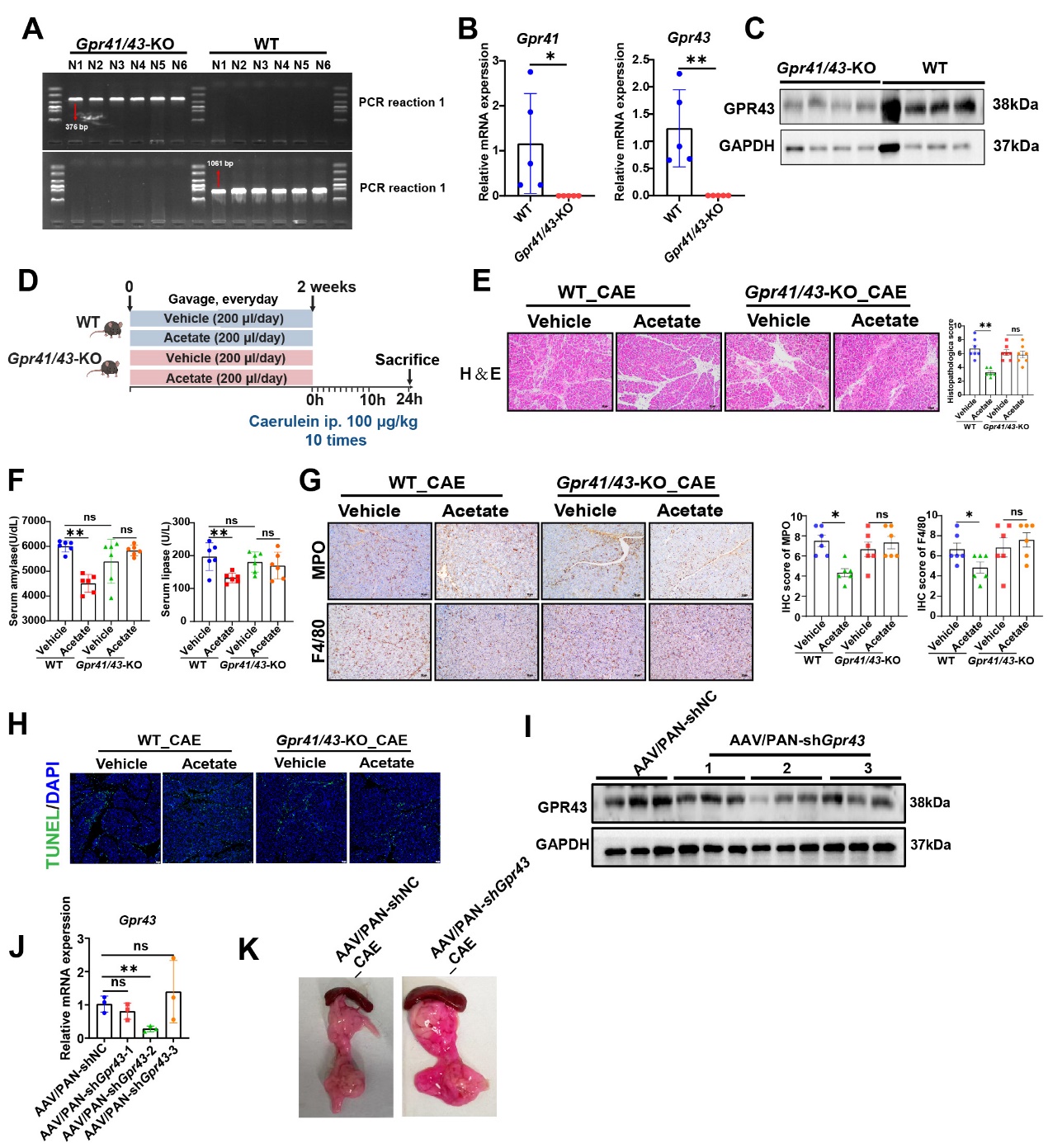


**Figure S7: GPR43 is required for acetate-mediated protection against AP in mice.** (A) PCR genotyping for the validation of *Gpr41/43*-KO efficiency in mice (n=6). (B–C) qRT‑PCR and Western blot analyses of *Gpr41* and *Gpr43* expression in pancreatic tissues from WT and *Gpr41/43*-KO mice (n=4–5). (D) Establishment of a CAE-induced AP mouse model in *Gpr41/43*-KO mice. (E) Representative images of pancreatic H&E staining and histopathological scores (n=6). (F) Serum amylase and lipase levels (n=6). (G) Representative images of IHC staining for MPO and F4/80 and their scores (n=5). (H) TUNEL staining of the pancreas. (I–J) Western blot and qRT‒PCR analyses of *Gpr43* expression in the pancreatic tissues of mice from three groups infected with distinct AAV/PAN-sh*Gp*r43 sequences (n=3). (K) Representative images of the pancreas after the injection of AAV/PAN-shNC or AAV/PAN-sh*Gpr43*. Scale bar: 10 μm. The two-sided p values were determined using one-way ANOVA with Dunnett’s multiple comparisons test (E–G, J), and the data are presented as the means ± SDs. **P* < .05, ***P* < .01, and ****P* < .001. IHC: immunohistochemistry.


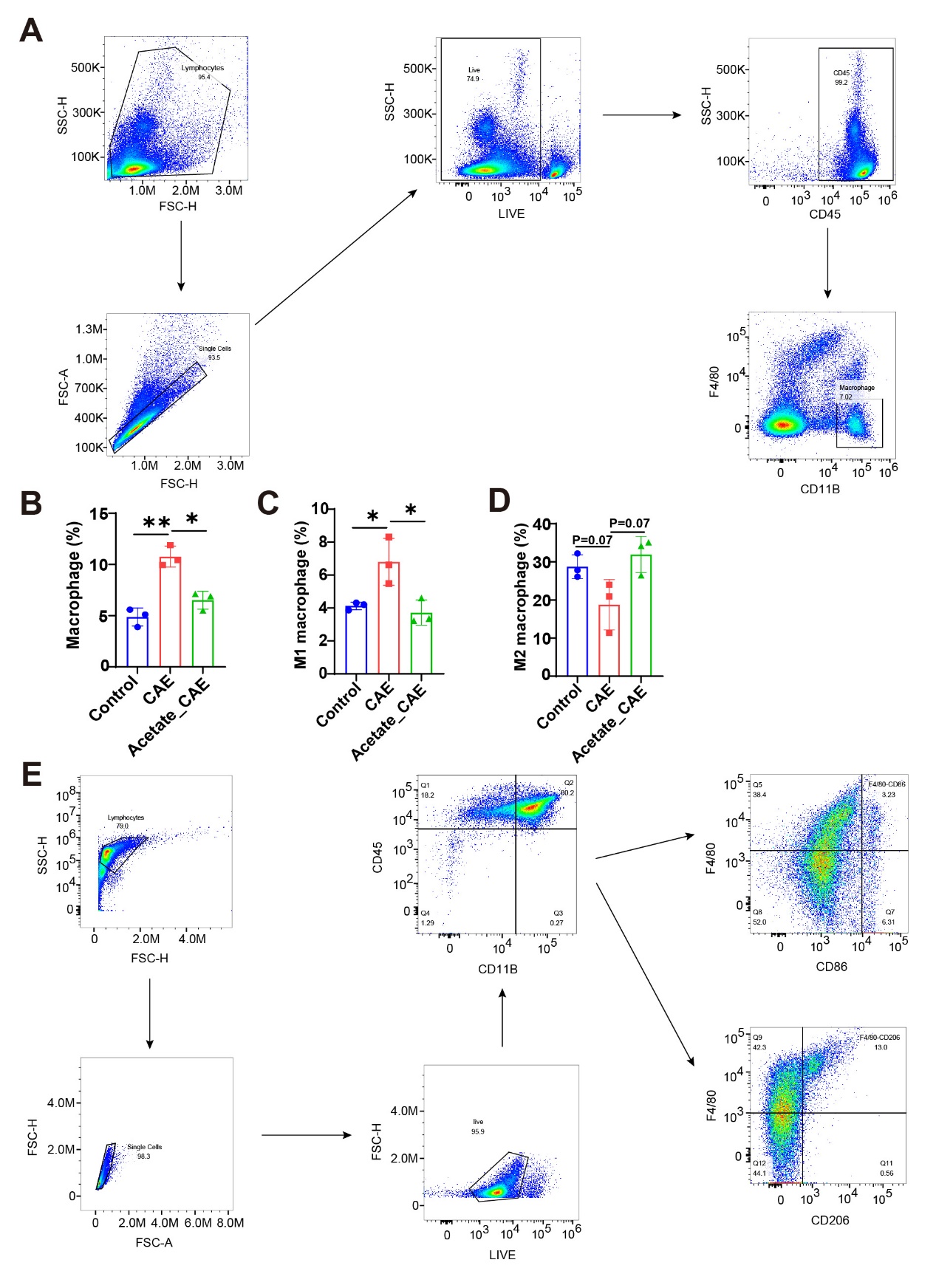


**Figure S8: Flow cytometry gating strategies for macrophage polarization.** (A) Gating strategy for macrophages. (B–D) Percentages of macrophages, M1 macrophages and M2 macrophages in spleen tissues (n=3). (E) Gating strategy used to assess macrophage polarization. The two-sided p values were determined using one-way ANOVA with Dunnett’s multiple comparisons test (B–D), and the data are presented as the means ± SDs. **P* < .05, ***P* < .01, and ****P* < .001.


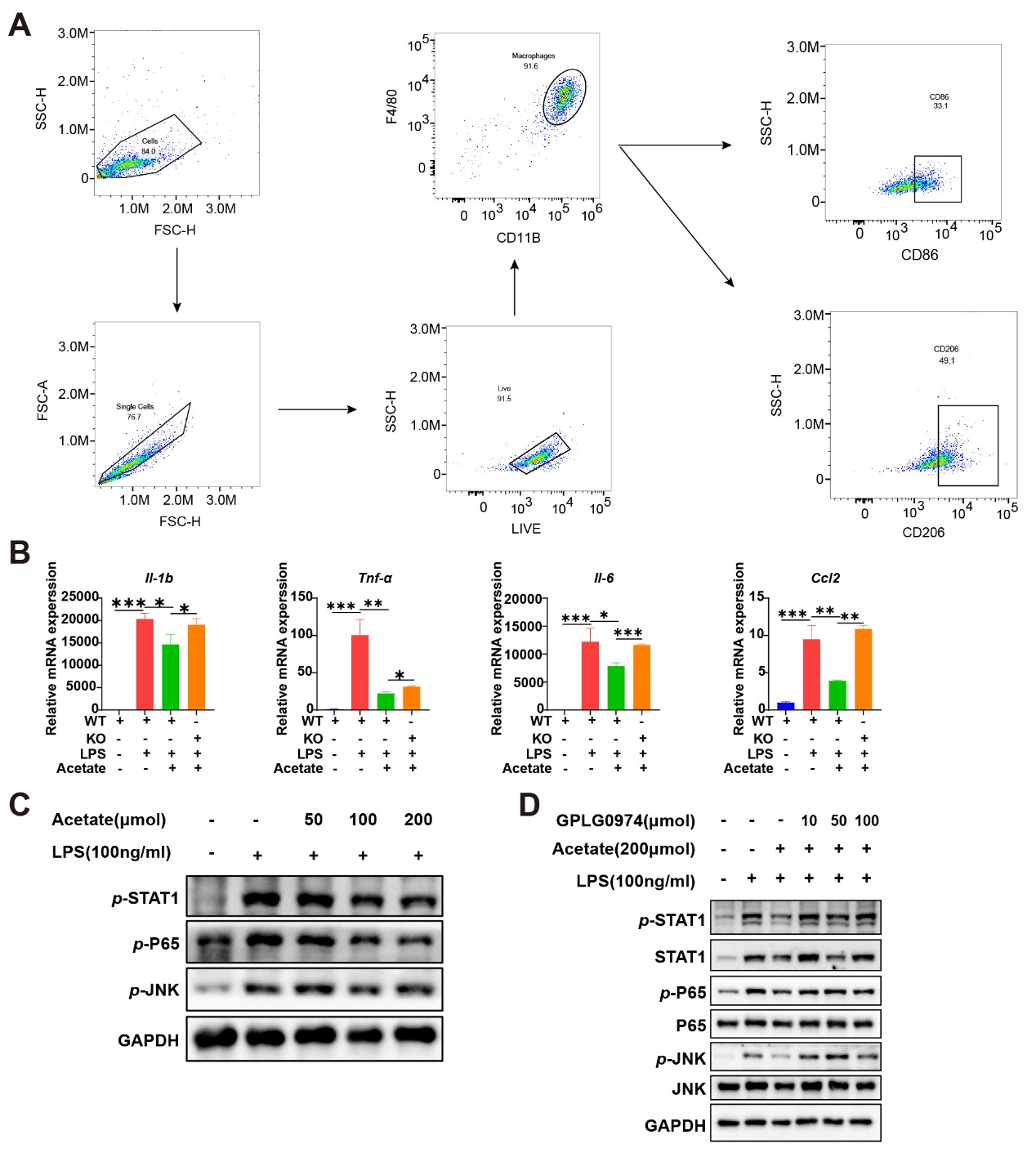


**Figure S9: GPR43‑dependent effects of acetate on macrophage polarization and inflammatory signaling. (**A) Gating strategy used to detect macrophage polarization in BMDMs. (B) Measurement of M1 macrophage markers by qRT‑PCR in BMDMs from *Gpr41/4*3-KO or WT mice stimulated with LPS (1 μg/ml) and/or acetate (200 μM) (n=3). (C) Western bloting analysis of p‑STAT1, p‑P65, and p‑JNK levels in RAW264.7 cells treated with LPS in the presence of acetate at the indicated concentrations (0, 50, 100, or 200 μM). (D) Western bloting analysis of p‑STAT1, p‑P65, and p‑JNK levels in LPS‑stimulated RAW264.7 cells cotreated with acetate (200 μM) and increasing concentrations of the GPR43 antagonist GLPG0974 (0, 10, 50, or 100 μM). The two-sided p values were determined using one-way ANOVA with Dunnett’s multiple comparisons test (B), and the data are presented as the means ± SDs. **P* < .05, ***P* < .01, and ****P* < .001.

**Table S1: Baseline characteristics of AP patients.**

**Table S2: Primer sequences used for qRT‑PCR.**

**Table S3: The primers used for mouse genotyping**
