# Supplementary material for: Bifidobacterium Pseudolongum‐Derived Acetate Attenuates Acute Pancreatitis Through GPR43‐Mediated Suppression of M1 Macrophage Polarization
Source: Adv Sci (Weinh). 2026 Mar 28;13(32):e17642. doi: 10.1002/advs.202517642 (PMC13252603; doi:10.1002/advs.202517642)
Supplement: Supplementary file 3 — Supporting file 3: advs75008‐sup‐0003‐Table S2.docx [file ADVS-13-e17642-s002.docx]

**Supplementary table 2 List of qRT-PCR primers used in this study**

| **Primer ID** | **Sequence (5’-3’)** |
| --- | --- |
| Mouse-*Gapdh*-F | TGACCTCAACTACATGGTCTACA |
| Mouse-*Gapdh*-R | CTTCCCATTCTCGGCCTTG |
| Mouse-*Il-6*-F | CTGCAAGAGACTTCCATCCAG |
| Mouse-*Il-6*-R | AGTGGTATAGACAGGTCTGTTGG |
| Mouse -*Il-1b*-F | GAAATGCCACCTTTTGACAGTG |
| Mouse-*Il-1b*-R | TGGATGCTCTCATCAGGACAG |
| Mouse-*Tnf-α*-F | CAGGCGGTGCCTATGTCTC |
| Mouse-*Tnf-α*-R | CGATCACCCCGAAGTTCAGTAG |
| Mouse-*Ccl2*-F | TAAAAACCTGGATCGGAACCAAA |
| Mouse-*Ccl2*-R | GCATTAGCTTCAGATTTACGGGT |
| Mouse-*Il-10*-F | CTTACTGACTGGCATGAGGATCA |
| Mouse-*Il-10*-R | GCAGCTCTAGGAGCATGTGG |
| Mouse-*Cd163*-F | GGTGGACACAGAATGGTTCTTC |
| Mouse-*Cd163*-R | CCAGGAGCGTTAGTGACAGC |
| Mouse-*Cd86*-F | TCAATGGGACTGCATATCTGCC |
| Mouse-*Cd86*-R | GCCAAAATACTACCAGCTCACT |
| Mouse-*Defa5*-F | TCAAAAAGCTGATATGCTATTG |
| Mouse-*Defa5*-R | AGCTACAGCAGAATACGAAAG |
| Mouse-*Defa21*-F | GAGAGATCTGATCTGCCTTTG |
| Mouse-*Defa21*-R | CAGCGCAAAAAAGGTCCTGC |
| Mouse-*Lysozyme1*-F | TACAACCGTGGAGACCGAAGCA |
| Mouse-*Lysozyme1*-R | TGGCTGCAGTGATGTCATCCTG |
| Mouse-*Gpr43*-F | CTTGATCCTCACGGCCTACAT |
| Mouse-*Gpr43*-R | CCAGGGTCAGATTAAGCAGGAG |
| Mouse-*Gpr41*-F | CTTCTTTCTTGGCAATTACTGGC |
| Mouse-*Gpr41*-R | CCGAAATGGTCAGGTTTAGCAA |
| Human-GAPDH-F | GGAGCGAGATCCCTCCAAAAT |
| Human-GAPDH-R | GGCTGTTGTCATACTTCTCATGG |
| Human-IL-6-F | ACTCACCTCTTCAGAACGAATTG |
| Human-IL-6-R | CCATCTTTGGAAGGTTCAGGTTG |
| Human-TNF-α-F | CCTCTCTCTAATCAGCCCTCTG |
| Human-TNF-α-R | GAGGACCTGGGAGTAGATGAG |
| Human-IL-1b-F | ATGATGGCTTATTACAGTGGCAA |
| Human-IL-1b-R | GTCGGAGATTCGTAGCTGGA |
| Human-CCL2-F | CAGCCAGATGCAATCAATGCC |
| Human- CCL2-R | TGGAATCCTGAACCCACTTCT |
